# Mode-resolved picosecond single-photon polarimetry maps modal dynamics in multimode fibers

**DOI:** 10.1038/s41467-026-72129-w

**Published:** 2026-05-07

**Authors:** Harikumar K. Chandrasekharan, Ross Donaldson

**Affiliations:** Scottish Universities Physics Alliance, Institute of Photonics and Quantum Sciences, School of Engineering and Physical Sciences, Heriot-Watt University, Edinburgh, Scotland UK

**Keywords:** Imaging and sensing, Ultrafast photonics

## Abstract

Polarization dynamics in multimode optical fibers (MMFs) play a key role in applications ranging from high-capacity communication to distributed sensing. However, most approaches probe only a single polarization state, limiting polarimetric characterization and leaving orthogonal dynamics hidden. Here, we show that time-resolved single-photon avalanche diode (SPAD) arrays enable quasi-real-time observation of dual-polarization mode dynamics in few-mode fibers. Simultaneous detection of orthogonal polarization channels provides picosecond time-of-flight polarimetric readout, revealing spatio-temporal correlations between LP_01_ and LP_11_ modes under stress. Building on this capability, we introduce a SPAD-based single-photon polarimetric platform that reconstructs Stokes vectors across  ~ 1000 spatial channels with 55 ps resolution, visualizing complex modal dynamics in MMFs. Through Hilbert-transform analysis, the system provides mode-resolved Stokes retrieval with per-pixel minimum detectable modulation of 0.002 (0.2%, 3*σ*) and signal-to-noise ratios up to 33 dB. This scalable platform enables ultrafast Stokes polarimetry in MMFs, opening new opportunities in classical and quantum photonics.

## Abstract (web summary)

Single-photon real-time imaging reveals the polarization dynamics of spatial modes in few and multimode optical fibres, enabling mode-resolved polarimetry and visualization of complex fibre dynamics.

## Introduction

Polarization-dependent mode behavior in optical fibers underpins applications in telecommunications^1,2^, sensing^3,4^, and imaging^5–7^. Single-mode fibers (SMFs) offer high-bandwidth, long-haul links and precise sensing via fundamental-mode confinement^8,9^, but deployment is costly due to alignment-sensitivity^10^, and performance remains bounded by nonlinear Shannon-limit considerations^11^. Multimode fibers (MMFs), with larger cores supporting many spatial modes, enable mode multiplexing and thus higher-capacity transmission at lower cost as well as easier installation^12,13^. However, their modal profiles vary with geometry, environment, and input polarization; perturbations induce mode mixing, dispersion, and interference that shape propagation and signal integrity^14–17^. Properly exploited and in some cases corrected for, these multimodal photon dynamics can advance communications, sensing, and endoscopic or deep-tissue imaging^18–20^. Accordingly, characterizing such dynamics across polarization states is essential for high-precision transmission, high-dimensional encoding, and imaging through scattering media^21,22^.

The high-fidelity spatial information of optical signals in MMF systems can be extracted through advancements in multiplexing, mode tomography, holography, single-photon detection, and machine learning techniques^23–26^. However, these techniques face several challenges and limitations, including mode crosstalk, computational complexity, sensitivity to alignment, and system cost, which hinder their widespread adoption. Furthermore, methods like machine learning require significant data and computational resources, while techniques such as digital holography demand precise optical alignment and often fail to resolve full polarization behavior at ultrafast timescales^27^. Critically, most existing approaches are limited in their ability to perform simultaneous, mode-resolved, time-resolved, and polarization-resolved measurements at the single-photon level —a capability essential for next-generation fiber-based quantum networks, time-resolved polarimetric imaging, and optical neural interfaces.

Regardless of the developed technology, a key capability for the future MMF characterization system is the real-time and precise calibration of photon dynamics in two orthogonal polarization states at timescales down to several picoseconds (ps) to nanoseconds (ns). Advancements in complementary metal-oxide-semiconductor (CMOS) technology have led to the development of sophisticated 2D single-photon avalanche detector (SPAD) arrays, achieving temporal resolutions as fine as the picosecond range. With their impressive ultrafast imaging capabilities, these SPAD array detectors have proven to be ideal for various real-world applications, such as fluorescence lifetime imaging microscopy^28^, time-of-flight imaging^29,30^, time-stretch imaging^31^, single-photon multimode imaging^32^, and quantum key distribution^33^.

Despite advances in detectors, ultrafast optical systems still lack integrated, mode- and time-resolved analysis—especially full-field Stokes measurements across applications. Conventional polarimeters such as DoFP^34^ and DoAP^35^ offer fast or full-Stokes acquisition but miss either spatial resolution, single-photon sensitivity, or the time resolution needed to resolve multimode structure. Recent MMF polarimetry also leaves gaps: camera-based speckle Stokes is frame-limited (ms-*μ*s)^36^; temporal/polarization control in MMFs omits mode-resolved Stokes^37^; and SNSPD full-Stokes systems are not mode-resolved in fibers^38^. Likewise, S_2_ imaging^39^, Mueller-matrix tomography^40^, and balanced detection^41^ often rely on sequential scanning or bulk optics, limiting spatial fidelity and frame rates.

In this paper, we present a silicon CMOS SPAD-2D-array-based system that enables simultaneous, 2D polarization-dependent mode dynamics and scan-free, mode-resolved Stokes extraction at the single-photon level across  ≈ 1000 spatial channels in a few-mode fiber (FMF). We further showcase system sensitivity in graded-index MMF, capturing subtle, spatially varying polarization modulations not accessible with conventional or camera-based polarimetry. As a first demonstration, we resolve the polarization-dependent evolution of FMF modal contents, capturing mode coupling under controlled and uncontrolled perturbations. We then use a rotating quarter-wave plate (QWP) to obtain spatially and temporally resolved Stokes measurements across 1000 ultrafast channels with 55-ps sampling. Applying a Hilbert-transform estimator rather than global fitting, we recover the full angle-resolved Stokes set in FMF and MMF. Critically, the system supports mode-resolved polarimetric imaging of LP_01_ and LP_11_ modes in FMF. Moreover, 55-ps time-resolved Stokes retrieval isolates modal signatures and polarization trajectories in MMFs with limited intermodal dispersion, showing sensitivity and robustness under weak delay contrast. The architecture achieves sensitivity up to 0.002 photons per channel and supports 2 ms per QWP angle acquisitions, enabling ultrafast, low-light MMF polarimetric imaging.

## Results

**Fig. 1:**
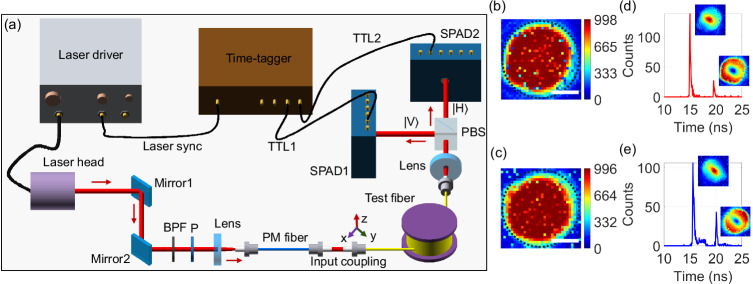
Dual-polarization light-in-flight spatial mode imaging system and SPAD-based detection scheme. **a** Experimental set-up for the dual-polarization light-in-flight spatial mode imaging system. At the two SPAD arrays, TCSPC measurement initiates when the signal from the fiber arrives at the SPAD pixels, and the periodic trigger signals from the time-tagger stop the photon measurement. BPF- bandpass filter, P- polarizer, PBS- polarizing beam splitter. **b**, **c** Topography of summed counts acquired in SPAD1 (**b**) and SPAD2 (**c**) for the full acquisition window. The dotted circle marks the approximate mode-imaging region on the arrays. Scale bars, 0.5 mm. **d**, **e** A single representative pixel (pixel 525) from each SPAD array with the TCSPC trace. The two peaks in each trace correspond to LP_01_ and LP_11_ modes, respectively. Summing the photon counts across each peak will allow the observation of polarization-dependent mode energy distributions on two SPAD arrays as represented in the inset images.

**Experimental set-up:** The initial focus of this work was to demonstrate ultrafast monitoring of spatial mode dynamics in an FMF supporting three distinct modes. The experimental setup used to observe dual-polarization mode dynamics in FMF is shown in Fig. 1. A pulsed laser (PicoQuant laser) with a pulse duration of 70 ps at full-width at half-maximum (FWHM) was used for ultrafast imaging of spatial modes. The repetition rate of the laser was set to 38 MHz, at an operating wavelength of 850 nm, with a bandpass bandwidth of  ± 2.5 nm. The average optical power at this rate was measured to be  ≈ 2 mW at the laser output aperture. A bandpass filter (BPF) with a 10 nm spectral bandwidth, designed to center at 850 nm, is utilized as an angle-tunable filter at the input to narrow the laser bandwidth. After inserting the BPF, the measured bandwidth is 0.48 nm at FWHM, with the central wavelength shifted to 852 nm. A polarizer (P) is used after the BPF to set the input polarization state and enforce the desired extinction ratio at injection. The narrow passband light was then directed onto a polarization-maintaining fiber (PMF)(P5-780PM-FC-2, Thorlabs) using free-space optics.

The polarization into the PMF was aligned such that at the output,  ≈ 30% light is detected in the vertical arm of a polarizing beam splitter (PBS), and  ≈ 70% light is detected in the horizontal arm of the PBS. This 30:70 extinction will enable us to quantify the polarization scrambling in the test fibers, as a polarization-maintaining test fiber is expected to preserve the extinction ratio along the transmission line. Light from the PMF is then coupled to the proximal end of a 2.4 km long SMF-28 fiber (Corning SMF-28) using fiber-fiber butt coupling, where input coupling is optimized by adjusting the fiber facet using an XYZ-microblock stage. At 852 nm, SMF-28 supports three linearly polarized (LP) spatial modes: LP_01_, LP_11*a*_, and LP_11*b*_, hence used as an FMF for the measurements. The distal end of the SMF-28 is imaged using a lens (*f* = 3.1 *mm*) and directed into the PBS, which separates the spatial modes into the two polarization states. At both arms of the PBS, two SPAD arrays are positioned at equal optical path lengths at the image planes, with SPAD array 1 (SPAD1) capturing the vertically polarized mode profile and SPAD array 2 (SPAD2) capturing the horizontally polarized mode profile. With the spectrally narrowed, linearly polarized launch, the FMF chromatic dispersion is suppressed, ensuring clear temporal separation between the modes.

The SPAD arrays (Photon Force Ltd) operate in a reversed start-stop configuration with 32 × 32 pixels arranged in a square grid and microlens arrays that increase the effective fill factor to  ~ 20%, enabling two-dimensional imaging. Each SPAD pixel has a  ≈ 7 μm diameter photosensitive area, a 50 μm pixel pitch, and operates over the 350-1050 nm wavelength range. Crucially, for our application, each pixel has a dedicated time-to-digital converter (TDC) for independent time-correlated single-photon counting (TCSPC) with a dynamic range of  ≈ 56 ns and timing bin duration of 55 ps^29,42^. Due to variability among the individual TDCs in each SPAD pixel, the pixelwise instrument response function (IRF) FWHM ranged from 123 ps to 398 ps for SPAD1 (253 ± 48 ps) and from 136 ps to 411 ps for SPAD2 (289 ± 42 ps) (see Methods for details). To ensure accurate alignment across both SPAD arrays for pixel-wise signal comparison, two identical tube-lens adapters with irises were mounted on the PBS, with their opposite ends threaded onto the CS-mount sensor regions of the SPAD arrays. In addition, before the PBS, a portion of the SMF-28 mode profile is redirected to a CMOS camera via a non-PBS component (not shown in Fig. 1a) for optical alignment and mode-magnification optimization.

**Time-of-flight spatial mode imaging:** Once the magnification is optimized, the mode profiles from both PBS arms were projected onto the SPAD arrays’ sensor area. The TCSPC measurements (see “Methods”) of the SMF-28 mode profiles in two polarization states were recorded using SPAD arrays over a 2 ms acquisition. The topography of total photon flux within the acquisition window for both detectors is presented in Fig. 1b, c, along with a single representative pixel histogram (pixel 525) for each detector (Fig. 1d, e). As shown in Fig. 1d, e, modal dispersion causes the two spatial modes to arrive at the detectors at different times, appearing as two distinct peaks. In all measurements, the time axis of the TCSPC traces is inverted to correctly represent the time-of-flight, as required by the reverse start-stop configuration of the SPAD arrays. For both polarization states recorded on the SPAD arrays, the two orthogonal orientations of the LP_11_ mode (LP_11*a*_ and LP_11*b*_) cannot be resolved due to their near-degenerate spatial and temporal characteristics. As a result, the recorded signal represents a superposition of both orientations, which is acceptable for the current analysis since their combined modal behavior is the primary quantity of interest. Future implementations could resolve the LP_11*a*/*b*_ orientations by inserting a simple correlation filter or phase mask.

For the specific SMF-28 fiber used, the modal dispersion between the LP_01_ and LP_11_ modes is measured to be  ≈ 5 ns. Summing the photon counts across the signal peaks enables precise monitoring of the mode profiles, as illustrated in the inset images. Even after applying peak shift corrections to both SPAD arrays, the arrival times of modes in SPAD2 remain delayed by  ≈ 500 ps compared to those in SPAD1. The artifact is electrical, primarily resulting from variability in the transmission properties of the RF cables carrying TTL synchronization signals, such as impedance mismatch or signal dispersion.

**Fig. 2:**
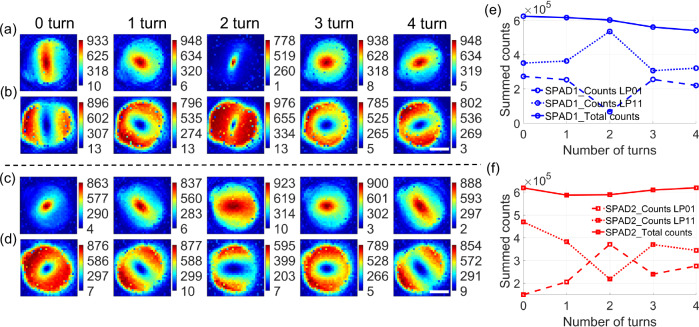
Polarization-resolved LP_01_ and LP_11_ mode dynamics under fiber perturbations. Optical intensity distributions for LP_01_ and LP_11_ modes recorded at SPAD1 (**a**, **b**) and SPAD2 (**c**, **d**) for different fiber conformations showcasing quasi-real time dual-polarization mode dynamics. All images cover the same spatial area, scale bar, 0.5 mm. **e**,** f** Summed photon counts for LP_01_ and LP_11_ modes showcasing energy flow between modes due to mode coupling.

**Mode dynamics under uncontrolled perturbations:** A set of experiments was conducted to demonstrate instantaneous ultrafast polarization mode dynamics using the dual-SPAD array system. First, the impact of uncontrollable fiber conformations on mode coupling and spatial energy distribution was investigated by coiling the fiber around a 12 mm diameter pipe at its input end, ~ 2 m from the input facet (see Supplementary Fig. S1a). Coiling the fiber can induce significant alterations in light propagation due to non-uniform strain distribution along respective fiber locations. Previous studies have extensively investigated stress-induced changes in refractive index and mode propagation, providing a deeper understanding of their effects^5,14,43–45^. Figure 2a–d presents single-photon, polarization-dependent modal dynamics across different fiber conformations, directly revealed by the dual-SPAD architecture, which records the vertical and horizontal states simultaneously and avoids temporal interleaving or drift. Note that the reflection arm of the PBS introduces a 180^∘^ phase shift to the image. To compensate for this, the image at the transmission end is rotated by the same amount, allowing for a pixel-wise comparison of the intensities between both images.

Due to uncontrolled bending, which prevents consistent conditions, the non-uniform stress and strain on the fiber cause unpredictable changes in mode coupling and energy distribution. This effect is evident in the “2-turn” case (Fig. 2a–d, column 3), where a significant discrepancy in mode energy distribution is observed between the two polarization states. To quantify these distributions, photon counts within each LP mode are summed and plotted as a function of turn number, as shown in Fig. 2e, f. Notably, despite significant energy flow between modes in the “turn” case, the total energy remains conserved. The experiment is then repeated for a total of 9 turns around the pipe, further quantifying the mode energy distribution, and the photon flux is plotted in Supplementary Fig. S2. Importantly, the total energy (SPAD1 + SPAD2) decreases with an increasing number of turns, a result of bend-induced loss—a well-known effect in optical fibers caused by evanescent photon leakage at bends^46^. These results highlight how mechanical perturbations such as bending can dynamically alter the orientation of spatial modes and redistribute energy between them. Such mode coupling effects are critical in applications where modal integrity and stability are essential, including mode-division multiplexing, polarization-multiplexed systems^47^, and fiber nonlinearity management for dispersion compensation^48^.

**Mode dynamics under controlled perturbations:** With precise control over induced deformations, the resulting mode dynamics can be harnessed for high-precision applications where the stability and reproducibility of these modal interactions are essential. To demonstrate the high degree of control over mode dynamics, we conducted a proof-of-concept experiment where fiber conformations were introduced in a more controlled manner. As shown in Supplementary Fig. S1b, the fiber was secured to an optical table, creating a high-strain bend along its length. A wooden cocktail stick (hereafter referred to as a cantilever) was then pressed against this high-strain section, with its position precisely controlled using an XYZ linear translation stage. We induced localized strain by systematically translating the cantilever forward along the fiber and observed its impact on mode profiles. The cantilever advanced 1 mm forward at each strain point, exerting increasing strain on the fiber. The energy flow and mode coupling across the 16 strain points are shown in Supplementary Fig. S3, with corresponding spatial mode energy distributions provided in the Supplementary Movie 1. Similar to the previous case, although inter-modal coupling induces multiple energy exchanges among modes, the total energy remains conserved. Another notable capability of our system is its ability to fully resolve and quantify intermodal energy flow at a single spatial point (pixel) across the fiber core, as illustrated in Supplementary Fig. S4. This enables single-point, in-core spatio-temporal tracking of intermodal coupling, providing direct insight into mode-to-mode energy dynamics with unprecedented spatial precision, benefiting photon-correlation research.

**Fig. 3:**
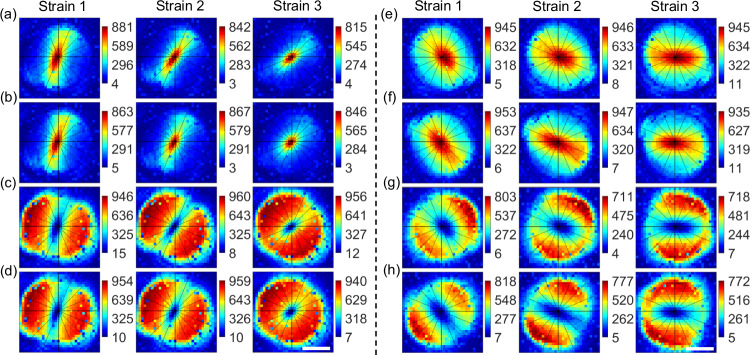
Polarization-resolved LP_01_ and LP_11_ mode evolution under controlled fiber perturbations. Intensity distribution of LP_01_ and LP_11_ modes for two polarization states recorded at SPAD1 (**a**–**d**) and SPAD2 (**e**–**h**) for three different cantilever positions showcasing controlled mode dynamics and energy flow. **a**, **b** LP_01_ mode distribution for three cantilever positions in forward (**a**) and reverse (**b**) positions for SPAD1. **c**, **d** Corresponding mode profile distribution for LP_11_ mode for forward (**c**) and reverse (**d**) positions. **e**, **f** LP_01_ mode distribution for three cantilever positions in forward (**a**) and reverse (**b**) positions for SPAD2. **g**, **h** Corresponding mode profile distributions for LP_11_ mode for forward (**g**) and reverse (**h**) positions. All images cover the same spatial area, scale bar, 0.5 mm. The minor dotted lines, spaced 22.5^∘^ apart, indicate the mode orientations for comparison. See Supplementary Movie 1 for energy flow and respective spatial mode profiles among two polarization states across 16 strain points.

The key capability of our demonstration is that these mode orientations can be reversibly controlled by repositioning the cantilever to its respective previous positions. As presented in Fig. 3, retaining the cantilever positions to the previous position results in obtaining the respective mode orientations. Negligible variations in mode orientations are observed in some profiles, which are likely due to manual positioning error of the cantilever during translation stage movements, as the mode profiles are highly sensitive to small strain variations. In practical applications, automated translational stages could provide precise and repeatable strain control to generate distinct mode orientations. Remarkably, for fibers supporting multiple spatial modes, the demonstrated technique has the potential to replace high-data-consumption electro-optic pattern generation devices. By generating well-distinguishable, multi-orientation mode profiles, it enables the creation of all-optical random patterns for high-speed compressive imaging^49,50^.

**Mode-resolved ultrafast Stokes polarimetry with FMF:** Simultaneous acquisition of polarization-dependent spatial energy distribution and mode coupling in optical fibers underpins advances in imaging, sensing, and high-capacity communications. To deliver true polarization-resolved functionality at scale, these platforms require complete modal decomposition—including full Stokes-vector extraction. Leveraging the 55-ps temporal resolution of each pixel in our SPAD arrays, we introduce a scan-free and scalable single-photon method for full spatio-temporal polarization analysis in  ≈ 1000 spatial points. To demonstrate this capability, we made no changes to the existing setup in Fig. 1 other than inserting a single additional optical element—a quarter-waveplate (QWP)—before the PBS. This simple addition enables full reconstruction of the Stokes vector at each pixel in both polarization-resolved detection arms, enabling mode-resolved, time-of-flight full-field Stokes polarimetry in MMFs with picosecond temporal sampling at the single-photon level.

To validate the generality and robustness of the technique, we applied it to two distinct fiber types: the FMF used in our earlier experiments and a highly MMF. As the first demonstration of our system’s full polarization-resolving power, we performed mode-resolved single-photon polarimetry with the FMF. Similar to previous measurements, the FMF is directly butt-coupled to the output of the PMF, ensuring that the input coupling conditions remained undisturbed. The respective mode profiles from both PBS arms were directed to the SPAD arrays, and the intensity distributions corresponding to the vertical (SPAD1) and horizontal (SPAD2) were recorded at 10 QWP rotation angles, from 0^∘^ to 90^∘^, spaced at 10^∘^ intervals, at the respective SPAD arrays. Unlike conventional polarimetry that sequentially scans analyzer angles, our fixed-PBS, dual-arm SPAD architecture acquires orthogonal polarizations in parallel, enabling scan-free, single-photon snapshots of modal evolution in real time. The topography of intensity distribution in both SPAD arrays within the selected regions for the 10 QWP angles is given in the Supplementary Figs. S5 and S6 for LP_01_ and LP_11_ modes. To ensure uniform angular response and suppress edge-related artifacts in the polarization analysis, a circular mask was applied to the SPAD array, centered at the array’s midpoint. This mask includes 932 pixels, representing the largest contiguous circular region fully contained within the sensor area.

**Fig. 4:**
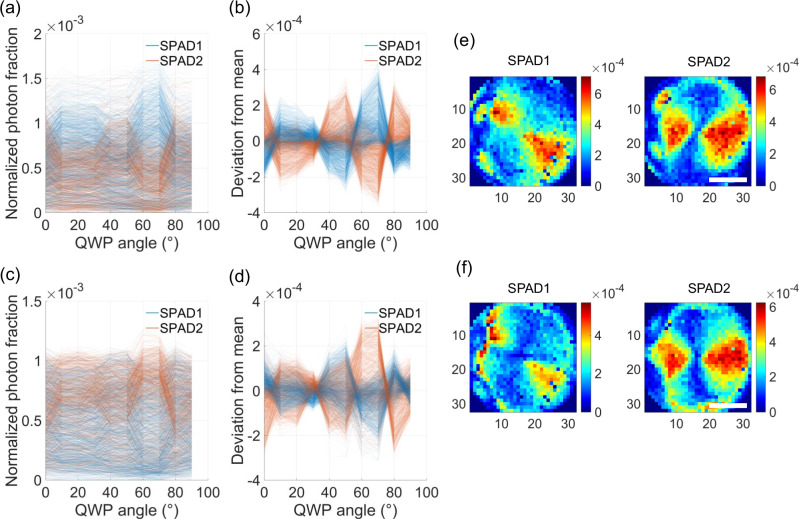
Pixel-resolved polarization modulation of LP_01_ and LP_11_ modes across SPAD arrays. **a**, **c** Normalized photon fractions recorded across 10 QWP angles for two orthogonal polarization channels (SPAD1 - Vertical: blue, SPAD2 - Horizontal: red), shown for the LP_01_ mode in (**a**) and LP_11_ mode in (**c**). Each trace corresponds to one of the 932 SPAD pixels per array. Panels (**b**) and (**d**) show the deviation of each pixel’s photon count from its mean across angles for the LP_01_ and LP_11_ modes, respectively, revealing the polarization modulation behavior. **e**, **f** Two-dimensional heat maps of modulation amplitude across the SPAD arrays for the LP_01_ and LP_11_ modes measured with SPAD1 and SPAD2, respectively, highlighting spatial variation in polarization sensitivity. Scale bar, 0.5 mm.

To visualize the pixel-wise response upon QWP-induced phase modulation, Fig. 4a, c represent normalized pixel-wise photon counts, plotted as a function of the QWP angle for both SPAD arrays for LP_01_ and LP_11_ modes. Fig. 4b, d represents the deviation of each pixel’s photon count from its mean across angles, revealing the polarization modulation. Panels (e) and (f) present 2D heat maps of modulation amplitudes from SPAD1 and SPAD2 pixels, highlighting spatial variations in polarization sensitivity. For the particular FMF used, although the polarization ratio after the PMF was measured to be 30:70 (V:H), the summed photon counts across 10 measurements remained nearly constant across all QWP angles, with  ≈ 49.5% light detected in the vertical arm and  ≈ 50.5% light detected in the horizontal arm, indicating high polarization scrambling in the FMF.

To extract the Stokes parameters from the time-resolved SPAD measurements, we employed a Hilbert-transform-based signal processing approach^51,52^. While Stokes parameters are conventionally obtained by fitting the intensity variations across QWP angles to sinusoidal or Fourier models, such methods yield only global fits and can obscure localized effects. In contrast, the Hilbert transform provides direct access to the instantaneous amplitude and phase of the horizontal and vertical components at each pixel and for every QWP angle. This enables a localized reconstruction of *s*_0_ − − *s*_3_ without relying on global curve fitting, making the method particularly suitable for spatially resolved fast polarization analysis under low-light, photon-limited conditions. This yielded the analytic signal representation for both H and V channels, from which we extracted the instantaneous amplitude and phase. The mean total intensity *s*_0_ was estimated from the sum of the two normalized channels. The differential amplitude and phase between the analytic V (SPAD1) and H (SPAD2) signals were then used to compute *s*_1_ and *s*_2_, while *s*_3_ was estimated from the average phase difference between the two channels as a proxy for circular polarization. This method enables robust estimation of the time-varying polarization state from overdetermined projections and is particularly well suited to real-time and low-light applications due to the high sensitivity of SPAD-based detection.

**Fig. 5:**
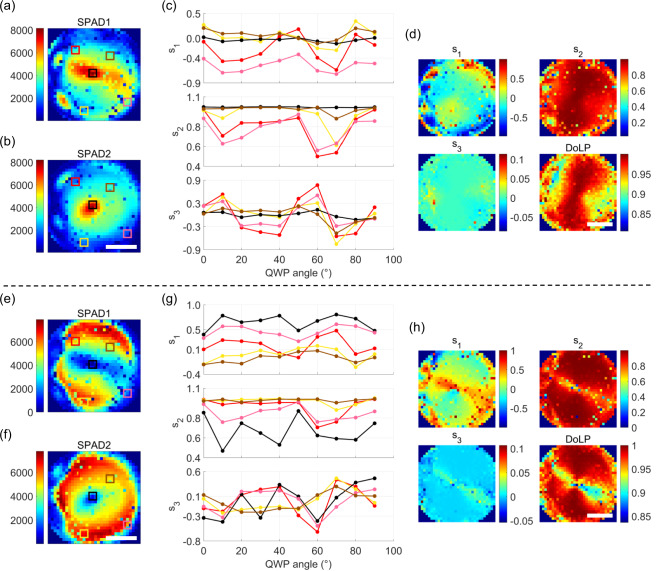
Stokes-vector reconstruction of LP_01_ and LP_11_ modes. Stokes vector analysis for LP_01_ (**a**–**d**) and LP_11_ (**e**–**h**).** a**, **b** Topography of summed photon counts over 10 QWP angles across the SPAD arrays. **c** Stokes vectors for 5 representative pixels (colored boxes in **a**, **b**). **d** Mean *s*_1_,  *s*_2_,  *s*_3_, and degree of linear polarization (DoLP), showcasing the global Stokes obtained from average values over 10 QWP angles. **e**–**h** Same analysis for LP_11_. Scale bars, 0.5 mm. See Supplementary Movie 2 for per-pixel Stokes reconstructions across all 10 QWP angles, illustrating the sequential evolution of the Stokes parameters over QWP angles.

These crucial experimental results are given in Fig. 5, which summarizes the mode-resolved Stokes analysis for LP_01_ and LP_11_ modes. For the LP_01_ mode (Fig. 5a–d), the representative pixels across various spatial co-ordinates show weak modulation with QWP angle, and the spatial maps remain uniform, but the mean Stokes traces reveal a clear offset in *s*_3_, indicating that the fundamental mode carries a stable elliptical (partly circular) polarization component across the profile. By contrast, in LP_11_ (Fig. 5e–h), different pixels exhibit distinct behaviors—some nearly flat, others strongly modulated with sign reversals in *s*_1_ or *s*_2_—yet the mean *s*_3_ remains close to zero. The maps confirm lobe-dependent variations in *s*_1_/*s*_2_ and a reduction in DoLP along the central node, pointing to spatially structured but predominantly linear polarization.

While Fig. 5d, h show the global Stokes values, the Hilbert analysis enables full angle-resolved pixel-wise Stokes evolution (see Supplementary Movie 2). The sequential Stokes reconstructions reveal that LP_11_ can exhibit localized, sign-opposed *s*_3_ at specific analyzer angles, which cancel in the spatial average and thus explain the near-zero mean *s*_3_ despite non-zero local circular content. They also show pixel-dependent modulation depth and phase in *s*_1_ and *s*_2_—often *π*-shifted between lobes—driving the structured maps observed in Fig. 5. By contrast, LP_01_ maintains a coherent ellipticity offset (non-zero *s*_3_) with only mild spatial scatter. These features are accessible only because the Hilbert transform retrieves per-pixel Stokes vectors at each QWP angle, which captures dynamic polarization states without averaging artifacts, whereas standard sinusoidal fitting yields only global averages and obscures such spatially anti-symmetric polarization.

**Fig. 6:**
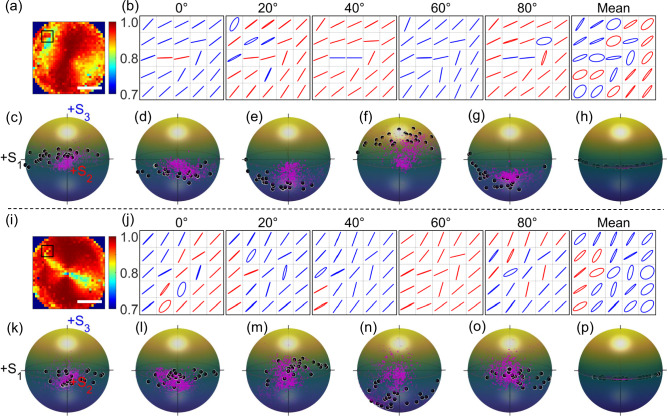
Polarization-state reconstruction of LP_01_ and LP_11_ modes using ellipse and Poincaré-sphere representations. Polarization characterization and Poincaré representation of the LP_01_ (**a**–**h**) and LP_11_ (**i**–**p**) modes in FMF.** a** Mean degree of polarization (DoP) across the 32 × 32 array for LP_01_; the black box indicates the selected 5 × 5 region for detailed analysis. Scalebar, 0.5 mm. **b** Polarization ellipses reconstructed for LP_01_ mode for each representative pixel in the selected region at QWP angles of 0^∘^, 20^∘^, 40^∘^, 60^∘^, 80^∘^, and the mean over all angles. Ellipse orientation and eccentricity encode the polarization azimuth and ellipticity, while color indicates handedness (blue = right-handed, red = left-handed). **c**–**h** Corresponding Poincaré spheres showing the distribution of Stokes vectors across the full array. Pink points represent all array pixels, while black markers highlight the representative pixels within the selected 5 × 5 region.** c**–**h** corresponds to the same QWP angles as in (**b**), and panel (**h**) shows the mean Stokes vectors over all angles, summarizing the overall polarization state of the mode. **i**–**p** Same analysis for LP_11_. Note that the mean Stokes points for LP_01_ (**h**) and LP_11_ (**p**) lie inside the Poincaré sphere because Euclidean averaging across angles reduces the degree of polarization.

To further substantiate our findings and highlight the capability of the approach, pixel-wise polarization states were reconstructed from the extracted Stokes parameters and mapped onto the Poincaré sphere, as shown in Fig. 6. A 5 × 5 pixel region of the array was selected to illustrate the dependence of the polarization state on the induced phase changes. The polarization ellipses for the representative pixels (black rectangular region) show nearly parallel orientation and consistent handedness for LP_01_, and their Poincaré markers cluster within a single region despite the finite *s*_3_ offset. For LP_11_, the representative pixels occupy separate regions of the sphere, with ellipses rotated between lobes and handedness flips, while the pink distribution of all pixels confirms broad spatial diversity.

Together, these results highlight a clear contrast: LP_01_ maintains a spatially uniform but elliptical polarization field, whereas LP_11_ exhibits lobe-dependent linear states with minimal circular content. This distinction illustrates how modal structure governs both the spatial uniformity and the form of polarization in FMFs. Beyond pixel-wise analysis, our approach supports partitioning the array into pixel subsets to evaluate collective modal behavior within each region. If a small lateral or rotational offset were present between the two SPAD arrays, corresponding pixels would probe slightly different parts of the modal field. Partitioning mitigates this by relying on regional averages rather than strict pixel-to-pixel correspondence. Because the modal fields vary smoothly across the fiber output, these regional averages remain stable under subpixel misalignments, effectively filtering out any residual chip displacement and ensuring robust Stokes-parameter retrieval.

A distinctive strength of our mode-resolved Stokes extraction technique is its scalability in resolving multiple guided modes. Numerical simulations were carried out to estimate the upper limit of temporally resolvable modes based on the SPAD timing performance (dynamic range of 56 ns and average IRF of 300 ps). The maximum number of resolvable modes is set by the delay spread between the slowest and fastest modes and the minimum temporal separation between adjacent modes. Strikingly, for a fiber with an 11 μm core radius and a refractive index contrast of 0.005, our method can resolve up to 41 distinct modes after 3.2 km of propagation through fiber at 852 nm. This fiber length was selected to maximize the usable modal delay spread while ensuring that the slowest mode remains within the 56 ns acquisition window. Beyond this range, later-arriving modes would fall outside the TCSPC window and wrap into the earliest bins, reappearing as full aliased peaks that overlap with the first-arriving modes and effectively merge those bins, scrambling the temporal-domain modal identification.

**Ultrafast Stokes polarimetry with MMFs:** In the heavily multimode regime, intermodal coupling defeats mode-resolved Stokes retrieval in our current scheme; however, quantifying polarization scrambling at an ultrafast temporal resolution remains crucial. Existing multimode speckle polarimetry is camera-based; the sensor frame rate (ms-*μ*s, exposure time) caps temporal resolution, preventing ultrafast (ns-ps)-resolved Stokes measurements^36,53^. To assess the applicability of our method for ultrafast multimode speckle polarimetry, we extended the measurements to an MMF (length- 1.2 km, graded index, 50 μm core diameter, NA 0.20) supporting 340 mode groups (680 individual LP modes, including polarization degeneracies), introduced into the setup by direct butt-coupling to the PMF output. Similar to the FMF case, the photon flux distribution at the PBS remained nearly balanced, with  ≈ 50% power in each arm (see Supplementary Fig. S7). Due to modal dispersion, the impulse response broadens to  ≈ 1 ns FWHM, accompanied by an extended tail. The pixel-wise fractional power and corresponding deviations from the mean photon counts are shown in Supplementary Fig. S8. Compared to the FMF, the deviation across both SPAD arrays is reduced by at least an order of magnitude, directly evidencing the highly depolarizing nature of the MMF. Remarkably, even under these strongly depolarizing conditions, the SPAD arrays reliably detected the weakly modulated photon signal. Supplementary Figs. S9 and S10 illustrate extracted Stokes vectors for selected pixels, including the polarization states of the representative region on the MMF core, highlighting the intricate scrambling characteristic of multimode propagation.

**Fig. 7:**
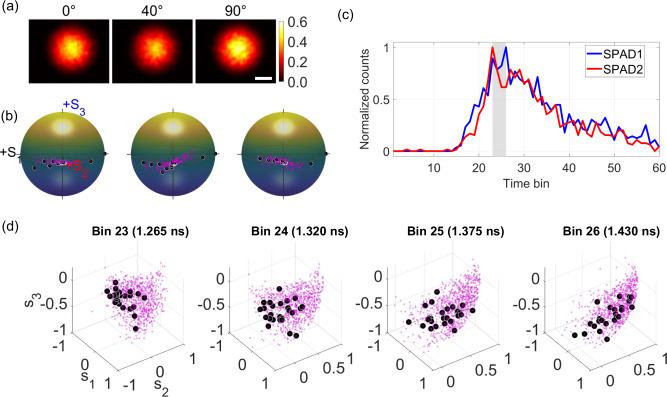
Ultrafast multimode speckle polarimetry and time-resolved Stokes analysis. **a** Camera images of the output mode field (before the PBS) at QWP angles 0^∘^, 40^∘^, and 90^∘^. Scale bar, 0.5 mm. **b** Corresponding Poincaré-sphere distributions of Stokes vectors across the full array; pink points show all pixels, black markers denote the representative 5 × 5 region. **c** TCSPC traces from the same representative pixel from both SPAD arrays after leading-edge alignment, used for bin-by-bin Stokes extraction. **d** Stokes distributions evaluated over four consecutive 55-ps bins (shaded window in **c**; 220 ps total) at QWP angle, 40^∘^. See Supplementary Movie 3 for the full scan across the TCSPC trace for QWP angles 0^∘^, 40^∘^, and 90^∘^.

Figure 7 summarizes the key results for the MMF measurements. Due to the large number of spatial modes, the QWP-induced phase distortions generated complex speckle patterns, as shown in Fig. 7a for QWP angles of 0^∘^, 40^∘^, and 90^∘^, recorded on a CMOS camera placed before the PBS (not shown in Fig. 1a). Consistent with earlier measurements, photon counts from both SPAD arrays were acquired for multiple QWP angles, and sequential Stokes parameters were extracted along the TCSPC trace. The corresponding Poincaré spheres, reconstructed from pixel-wise SPAD intensities in both polarization arms, are shown in Fig. 7b, directly visualizing the strong polarization scrambling characteristic of multimode propagation.

While Fig. 7b showcases the Poincaré representation across the full 1 ns signal trace, providing only an average view of modal scrambling, the true strength of our system lies in its ability to resolve Stokes parameters with 55-ps temporal resolution across the entire signal, without requiring complex post-processing. This capability moves beyond averaged polarization states and enables direct access to the ultrafast evolution of multimode polarization dynamics. As shown in Fig. 7c, each pixel-resolved TCSPC trace encodes the modal state corresponding to specific spatial coordinates in the fiber, from which a full vectorial representation of the mode evolution can be reconstructed with 55-ps precision. To illustrate this, Fig. 7d presents the Stokes vectors for QWP angle 40^∘^ within a 220-ps window (four time bins, shaded in Fig. 7c), where the values exhibit striking temporal fluctuations due to modal scrambling. A comprehensive view of these bin-by-bin Stokes dynamics is provided in Supplementary Movie 3, across the full signal window for three QWP angles. In Movie 3, the distinct pixel trajectories arise because each pixel records a different superposition of multimode fields with distinct group delays. As the 55-ps analysis bin advances, the relative modal weights and phases entering that bin change, shifting the local intensity and rotating the corresponding Stokes vector on the Poincaré sphere (see Supplementary Note 4 for details).

The bin-resolved Stokes retrieval presented here illustrates the core capability of our system but does not yet account for timing jitter from IRFs in the recorded signal traces. Future comprehensive processing—e.g., deconvolution with the measured IRFs and parameter estimation via a *χ*^2^ fitting procedure—should more reliably recover the underlying signal^54^. The modest temporal broadening observed in our test MMF reflects its graded-index profile, which is designed to suppress modal dispersion; a step-index MMF would be expected to exhibit a substantially broader temporal window. Overall, our approach enables direct observation of full-field, ultrafast polarization dynamics in MMFs with 55 ps sampling, supporting advances in fiber sensing, secure communications, and time-resolved polarimetric imaging.

**Sensitivity of the picosecond polarimetry system:** The sensitivity of our ultrafast polarimetry is quantified as the minimum detectable modulation (MDM), as the initial attempts to calculate the MDM and signal-to-noise ratio (SNR) using signal strength versus noise measured outside the signal region resulted in artificially high SNR values and unrealistically low MDM due to the negligible noise floor. For this, each SPAD array was flat-illuminated by the PMF output passed through a fixed linear polarizer (no beamsplitter), ensuring identical polarization and flux without inter-arm equalization. For each pixel in the 32 × 32 SPAD arrays, the standard deviation of these measurements (background corrected) per pixel was calculated to quantify the variability in the relative photon response. By scaling this variability by 3 (corresponding to a 3*σ* confidence level), the MDM was determined, representing the smallest polarization-induced signal change that can be reliably detected above noise. This fractional MDM therefore provides a detailed pixel-wise map of the system’s polarimetric sensitivity (see Supplementary Figs. S11 and S12, Supplementary Note 5 for details).

After outlier removal, the two SPAD arrays showed comparable sensitivity: per-pixel MDM ranged from 0.0023-0.030 (SPAD1) and 0.0016-0.023 (SPAD2), with array-averaged MDMs of 0.013 and 0.0125, respectively. This corresponds to per-pixel SNRs of 31- 19 dB (SPAD1) and 33-21 dB (SPAD2). Consequently, the system resolves modulation as small as 0.2% on single pixels while maintaining an average sensitivity of 1.3% across the arrays. We also quantified per-pixel stability (coefficient of variation (CV)) and temporal drift from the same repeated measurements; see Supplementary Figs. S13 and S14 and Supplementary Note 6 for methodology and full results (low CV and negligible drift across both arrays).

For applications demanding exquisite sensitivity to photon modal dynamics—such as high-dimensional quantum entanglement—analysis can be confined to the highest-sensitivity pixels, delivering sub-percent modulation detection on the relevant modes while preserving 55-ps temporal resolution. Typical spectro-polarimetric systems achieve spectral ranges on the order of  ≈ 0.01-0.1 nm (resolving power *R* ≈ 10^−4^ − 10^−5^ nm) using long exposures or sequential modulation^55^. In contrast, with a temporal sampling resolution of 55-ps (corresponding to a Nyquist-limited spectral span of  ≈ 0.022 nm at 852 nm) and a 56 ns observation window (which sets the effective spectral resolution of  ≈ 4 × 10^−5 ^nm), our system enables full-field, high-resolution, time-resolved Stokes polarimetry in MMFs far exceeding standard techniques.

## Discussion

In this work, we demonstrate an efficient method for observing, inducing, and calibrating dual-polarization mode dynamics in MMFs using multipixel silicon CMOS SPAD 2D array detectors. Such strain-induced, controlled modal-energy localization, together with polarization-dependent mode coupling, enables impulse-response control in MMF systems^37^ and improves polarization-dependent coupling efficiency in optical links—particularly free-space, single-photon QKD—where the signal-spot size strongly limits the achievable secret-key rate^56^. This approach paves the way for compact, polarization-sensitive MMF characterization without complicated optical systems or computational requirements, and with further optimization can extend to fibers supporting up to 41 spatial modes, broadening applicability to mode- and polarization-dependent quantum entanglement^57,58^ and beyond.

Furthermore, we demonstrate full-vectorial, time-resolved Stokes reconstruction in a multimode system with 55-ps temporal resolution over a 56 ns dynamic range (effective spectral resolution 4 × 10^−5 ^nm), underscoring the architecture’s scalability and versatility. Simultaneous access to ultrafast polarization dynamics, spatial mode structure, and sub-percent photon sensitivity provides a powerful diagnostic for modal interference, mode coupling, and polarization scrambling. The platform is well-suited to studies of spatio-temporal correlation in multimode delivery^59^, polarimetric sensing for long-haul communications^60^, localizing polarization events along MMF fiber links for environmental monitoring^61^, and for isolating birefringent features under motion in fiber polarimetric endoscopy^62^.

Beyond physics, the dual-polarization imaging system has strong potential in biology: many specimens exhibit sensitivity to small changes in polarization arising from ordered structure, birefringence, or polarization-dependent scattering. Examples include collagen-rich tissues, myelinated nerve fibers, and amyloid fibrils^63–65^. Critically, ultrafast MMF-speckle polarimetry is natively compatible with single-ended (reflection) operation when the distal end is inaccessible; in this geometry, fiber perturbations transduce into robust Stokes signatures, enabling fast, accurate shape sensing—for example, real-time surgical-probe path identification in image-guided interventions^66^.

## Methods

### TCSPC Experimental details

As shown in Fig. 1a, the experimental setup employs two identical SPAD arrays placed at the two output ports of the PBS. The arrays are operated in a reverse start-stop configuration, in which photon arrivals from the test fiber serve as the start signals, while periodic laser triggers (matched to the laser repetition rate) define the stop events. The electrical trigger output of the laser (NIM signal) is routed to a time-tagger module (ID900, ID Quantique), which converts the signal into TTL logic levels and distributes it as the stop signals to both SPAD arrays. To mitigate saturation and photon pile-up^67^, we limited the per-pixel detection rate to  ≈ 10% of the pulse rate (1 photon detected in every 10 pulses).

In reverse start–stop mode, the TDC reports the interval *Δt* between a photon start and the next stop. The photon arrival time within the laser period is recovered as

1 $${t}_{{{\rm{arr}}}}={T}_{{{\rm{rep}}}}-\Delta t$$

with values wrapped to the interval [0, *T*_rep_), accumulated into the per-pixel histogram *H*_*i*_[*n*].

As part of the basic characterization of the SPAD arrays, we measured the IRF of every pixel in both detectors. The IRF captures the overall temporal broadening of the system, incorporating contributions from the excitation pulse duration, detector electronic jitter, and timing jitter introduced by RF cables and associated instrumentation. The arrays were uniformly illuminated with a narrow-band pulsed laser at 852 nm delivered through the PMF, and the FWHM of each pixel’s TCSPC histogram was extracted. The resulting IRF maps are presented in Supplementary Fig. S15, which shows the spatial topography of the FWHM across both arrays, along with representative traces for the pixels exhibiting the minimum and maximum IRF, and a simulated IRF corresponding to the average FWHM over the arrays. In addition, per-pixel TDC variations led to non-uniform timing offsets in the raw traces; we therefore determined the peak arrival time for each pixel, converted these offsets to integer multiples of the 55-ps bin, and used them to align all TCSPC traces to a common temporal reference on both arrays.

For the FMF results presented, counts were integrated in fixed windows around the LP_01_ and LP_11_ peaks to obtain modal energies. For the MMF measurements, a leading-edge signal alignment strategy was adopted, which also accounts for delays from polarization-mode dispersion; Stokes parameters were then computed bin-by-bin along the trace, yielding 55 ps temporal resolution.

### Hilbert transform–based stokes vector extraction

Two SPAD arrays were placed at the output ports of a fixed PBS: array 1 recorded the vertically polarized component *I*_*V*_ and array 2 recorded the horizontally polarized component *I*_*H*_. For each QWP rotation angle *θ*_*k*_ ∈ {0^∘^, 10^∘^, …, 90^∘^} (*k* = 1, …, *K*,  *K* = 10), photon count distributions were acquired from both arrays.

*Angle-resolved signals*.

Let *I*_*H*_(*i*, *j*, *θ*_*k*_) and *I*_*V*_(*i*, *j*, *θ*_*k*_) denote the measured intensities at pixel (*i*, *j*) for the *k*-th QWP angle. We define the sequences

$${h}_{ij}[k]:={I}_{H}(i,j,{\theta }_{k}),\,{v}_{ij}[k]:={I}_{V}(i,j,{\theta }_{k}),\,k=1,\ldots,K.$$

*Hilbert transform and phase retrieval*.

For each pixel (*i*, *j*), we form the analytic signals along the *angle index* using the discrete Hilbert transform ${{{\mathcal{H}}}}_{d}\{\cdot \}$:

2 $${\widetilde{h}}_{ij}[k]={h}_{ij}[k]+i\,{{{\mathcal{H}}}}_{d}\,\{{h}_{ij}[k]\},\,{\widetilde{v}}_{ij}[k]={v}_{ij}[k]+i\,{{{\mathcal{H}}}}_{d}\,\{{v}_{ij}[k]\}.$$

The instantaneous phases are

3 $${\phi }_{H}[i,j,k]=\arg \left({\widetilde{h}}_{ij}[k]\right),\,{\phi }_{V}[i,j,k]=\arg \left({\widetilde{v}}_{ij}[k]\right),$$

which are unwrapped along *k* to remove 2*π* discontinuities. The relative phase is then

4 $$\Delta \phi [i,j,k]={\phi }_{H}[i,j,k]-{\phi }_{V}[i,j,k].$$

*Stokes parameter estimation*.

The Stokes parameters are computed as

5 $${S}_{0}[i,j,k]={h}_{ij}[k]+{v}_{ij}[k],$$

6 $${S}_{1}[i,j,k]={h}_{ij}[k]-{v}_{ij}[k],$$

7 $${S}_{2}[i,j,k]=2\sqrt{{h}_{ij}[k]\,{v}_{ij}[k]}\,\cos \left(\Delta \phi [i,j,k]\right),$$

8 $${S}_{3}[i,j,k]=2\sqrt{{h}_{ij}[k]\,{v}_{ij}[k]}\,\sin \left(\Delta \phi [i,j,k]\right),$$

with normalized Stokes parameters

9 $${s}_{m}[i,j,k]=\frac{{S}_{m}[i,j,k]}{{S}_{0}[i,j,k]},\,m\in \{1,2,3\}.$$

## Supplementary information

Supplementary Information

Description of Additional Supplementary Files

Supplementary Movie 1

Supplementary Movie 2

Supplementary Movie 3

Transparent Peer Review file

## Data Availability

The data that support the findings of this study are openly available in the Heriot-Watt University PURE research data management system at 10.17861/2b4b1d77-177d-4935-a1bc-f709099cd42c.
