# Supplementary material for: Mode-resolved picosecond single-photon polarimetry maps modal dynamics in multimode fibers
Source: Nat Commun. 2026 May 7;17:6174. doi: 10.1038/s41467-026-72129-w (PMC13365444; doi:10.1038/s41467-026-72129-w)
Supplement: Supplementary file 1 — Supplementary Information [file 41467_2026_72129_MOESM1_ESM.pdf]

*Supplementary Material*

# Mode-resolved picosecond single-photon polarimetry maps modal dynamics in multimode fibers

Harikumar K Chandrasekharan[1,*] and Ross Donaldson[1]

[1]Scottish Universities Physics Alliance, Institute of Photonics and Quantum Sciences, School of Engineering and Physical Sciences, Heriot-Watt University, David Brewster Building, Edinburgh EH14 4AS, Scotland, UK

(*Corresponding Author; Email: hk47@hw.ac.uk)

## Supplementary Note 1: Uncontrolled and controlled fiber conformations

For the uncontrolled mode dynamics studies, a 12 mm Thorlabs post is used. The optical fiber is wrapped around this post, as illustrated in Supplementary Figure S1a. TCSPC measurements are then taken for different numbers of turns to analyze the resulting dynamics. For controlled mode dynamics studies, a cantilever is securely mounted on a XYZ translational stage. The optical fiber is carefully brought into controlled contact with the cantilever by precisely translating the stage, as depicted in Supplementary Figure S1b.

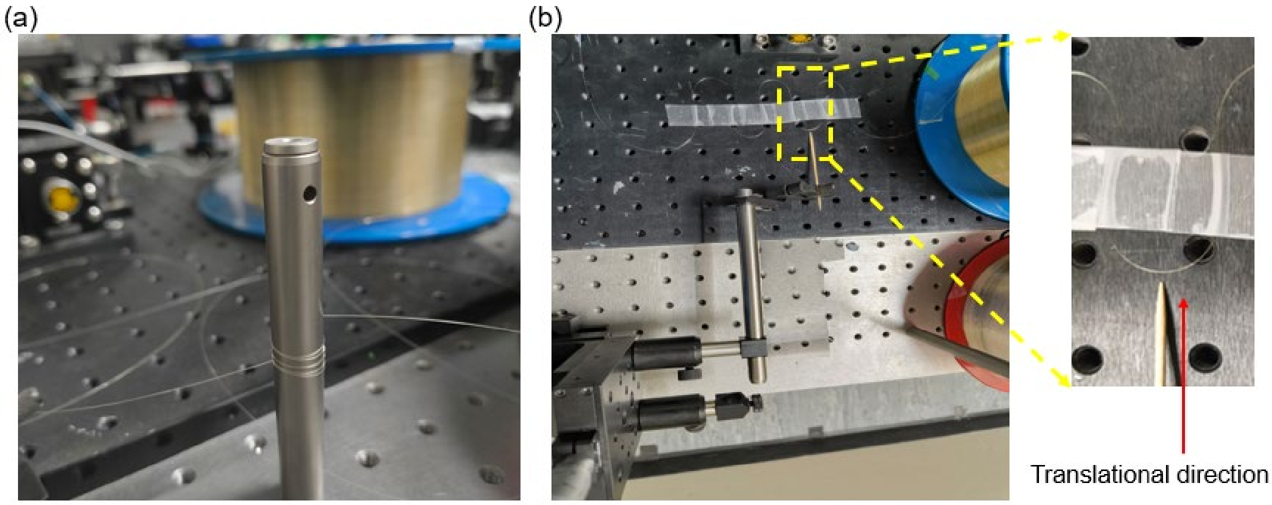


**Supplementary Figure S1: Experimental configurations for fiber perturbations.** Uncontrolled (a) and controlled (b) fiber conformations for the mode dynamics measurements.

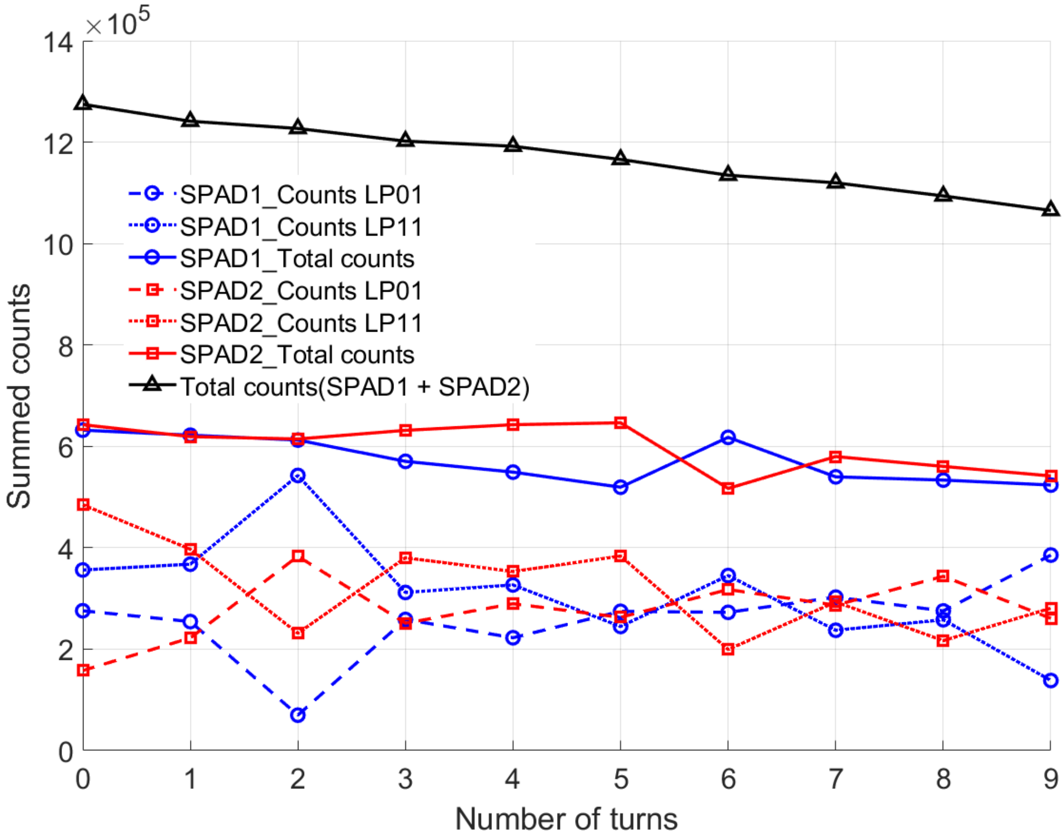


**Supplementary Figure S2: Inter-modal energy transfer under increasing fiber turns.** Summed photon counts over 9 measurements, demonstrating inter-modal energy transfer. The overall energy decreases with increasing turns, attributable to evanescent photon leakage.

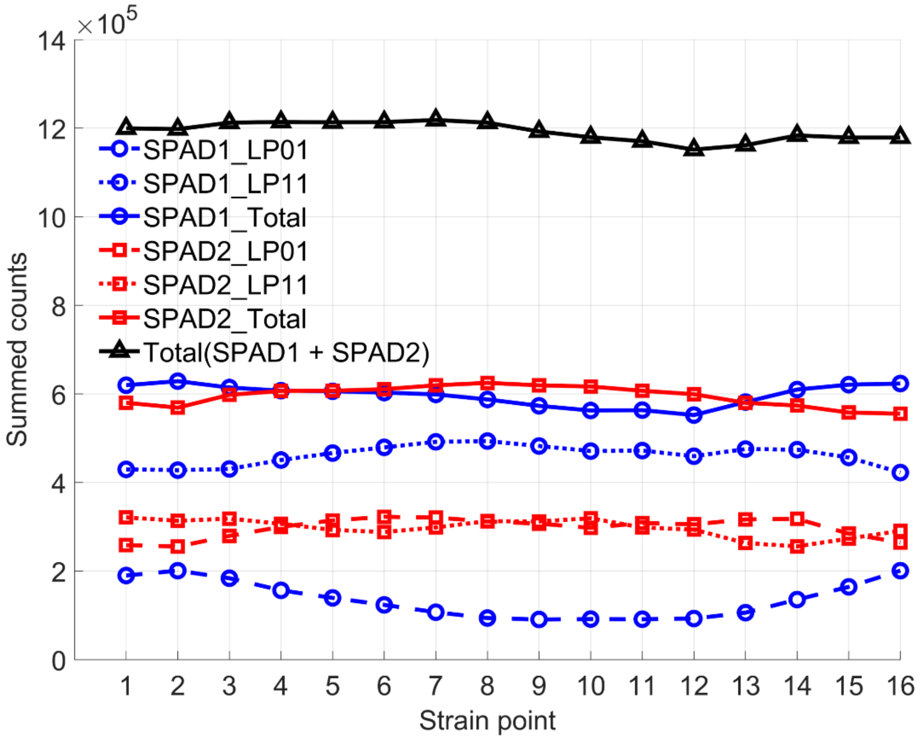


**Supplementary Figure S3: Inter-modal energy transfer across controlled strain points.** Summed photon counts among 16 strain points, demonstrating inter-modal energy transfer. The respective spatial energy distribution is provided in Supplementary Movie 1.

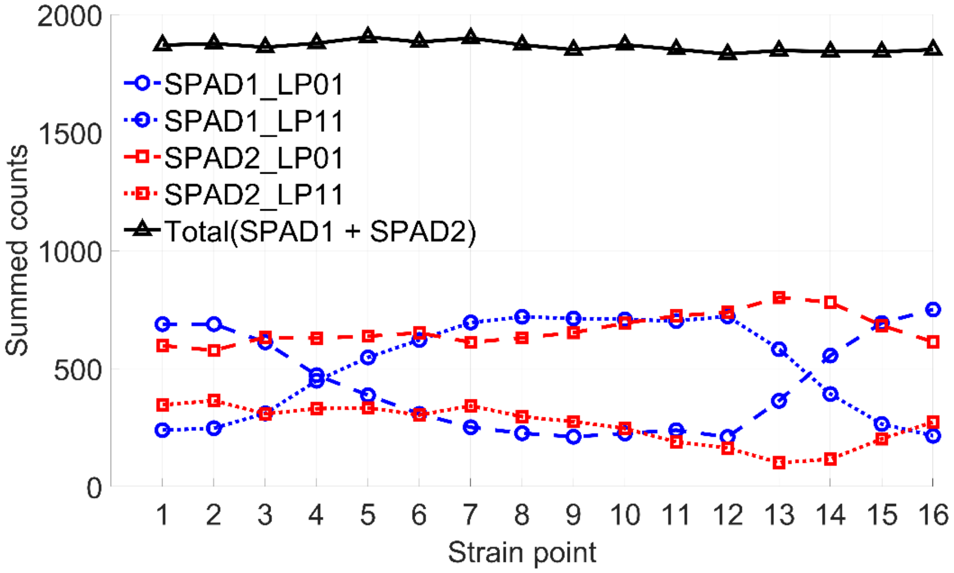


**Supplementary Figure S4: Inter-modal energy transfer at a representative spatial pixel.** Summed photon counts for the same spatial pixel (pixel 525) recorded on SPAD1 and SPAD2 across 16 strain points, illustrating modal energy redistribution at a single spatial location.

**Supplementary Note 2: Mode-resolved ultrafast Stokes polarimetry with FMF**

For the mode-resolved polarimetry measurements, the output field was routed through a quarter-wave plate (QWP) and a polarizing beam splitter (PBS), then imaged onto two SPAD arrays at the PBS's vertical (SPAD1) and horizontal (SPAD2) ports. Spatial maps of photon count for both modes across 10 QWP angles are shown in Supplementary Figures S5 and S6.

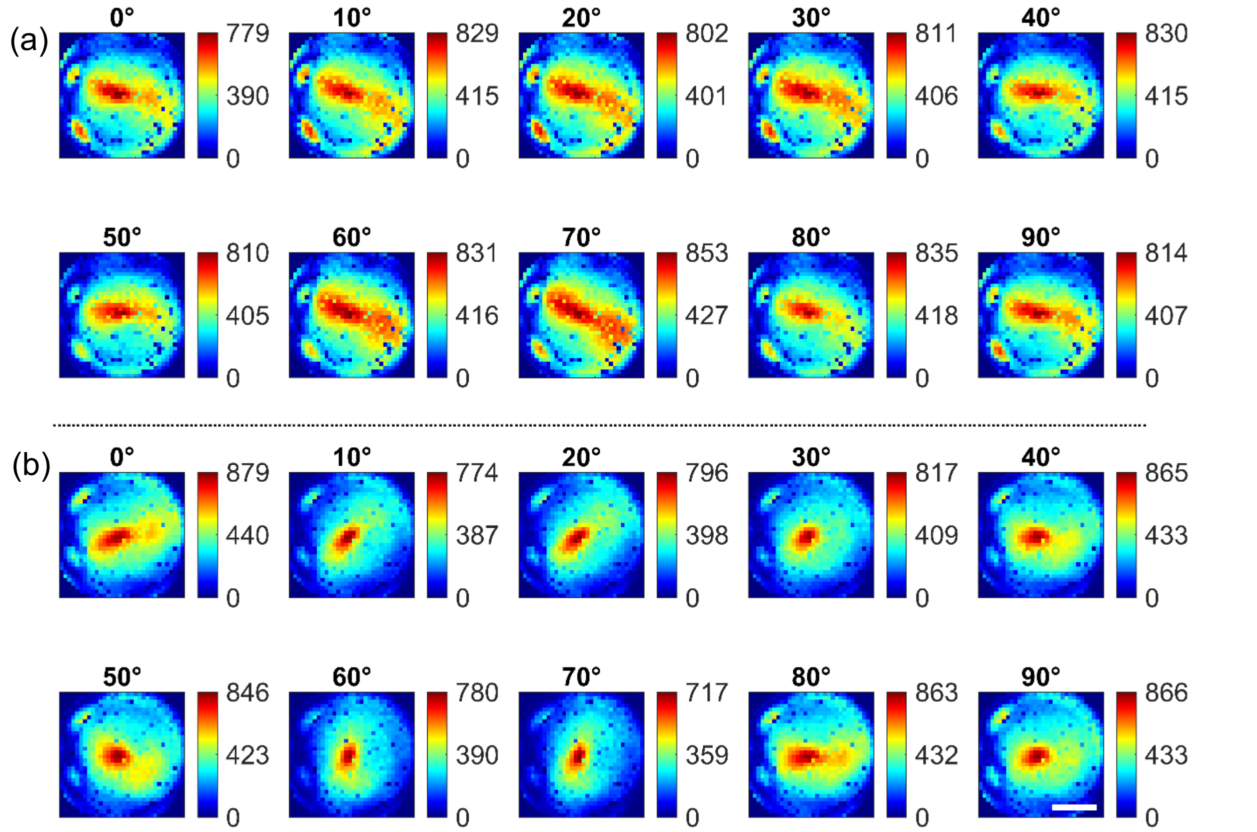


**Supplementary Figure S5: Topography of summed photon counts across QWP angles for the $LP_{01}$ mode.** Topography of summed photon counts across 10 QWP angles for SPAD array 1 (a) and SPAD array 2 (b) for $LP_{01}$ mode. Scale bar, 0.5 mm.

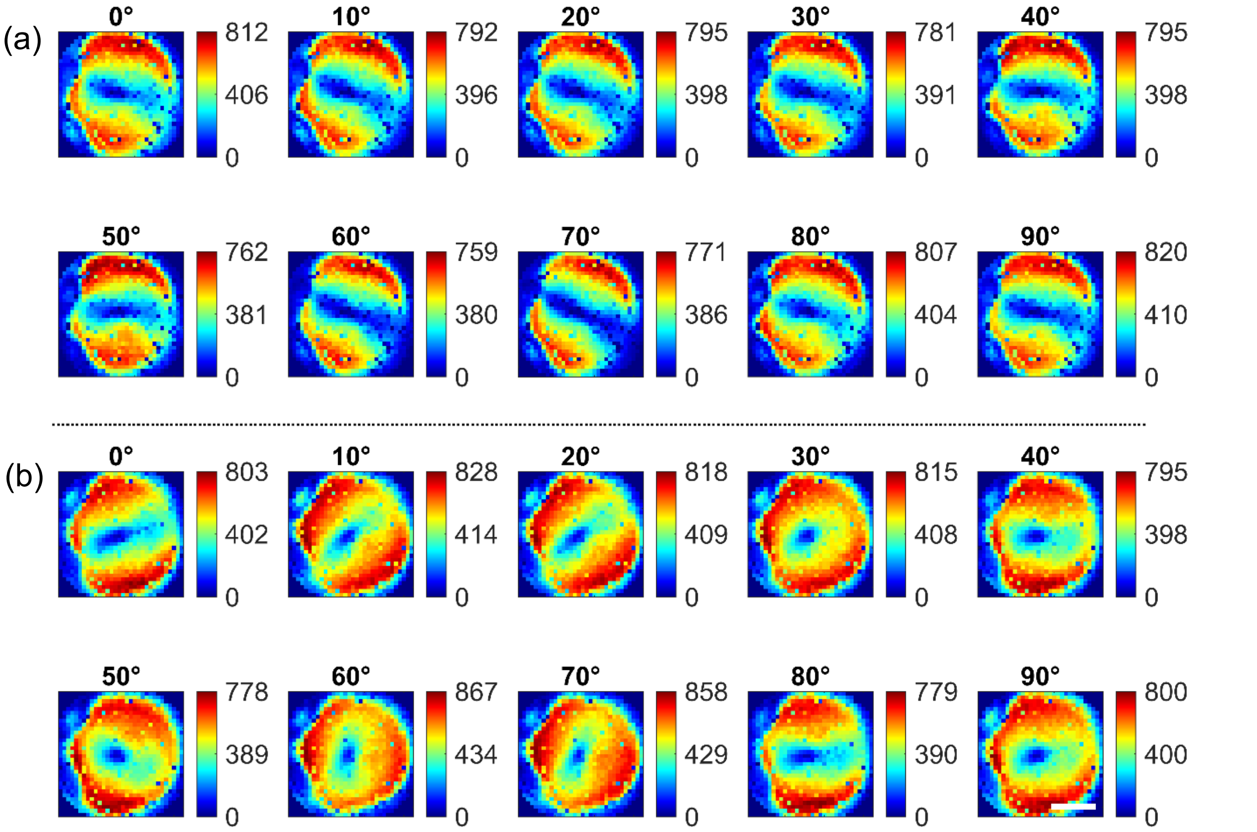


**Supplementary Figure S6: Topography of summed photon counts across QWP angles for the $LP_{11}$ mode.** Topography of summed photon counts across 10 QWP angles for SPAD array 1 (a) and SPAD array 2 (b) for $LP_{11}$ mode. Scale bar, 0.5 mm.

**Supplementary Note 3: Ultrafast Stokes polarimetry with MMF**

For the MMF polarimetry measurements, the output mode field was passed through the QWP and PBS and imaged onto two SPAD arrays placed at the PBS's vertical (SPAD1) and horizontal (SPAD2) ports. Photon counts for the SPAD arrays were recorded for ten QWP angles and presented in Supplementary Figure S7. Notably, with a 30:70 (V: H) launch state, the two arrays register nearly equal counts, indicating strong depolarization at the output—consistent with extensive intermodal coupling among the many modes supported by the MMF.

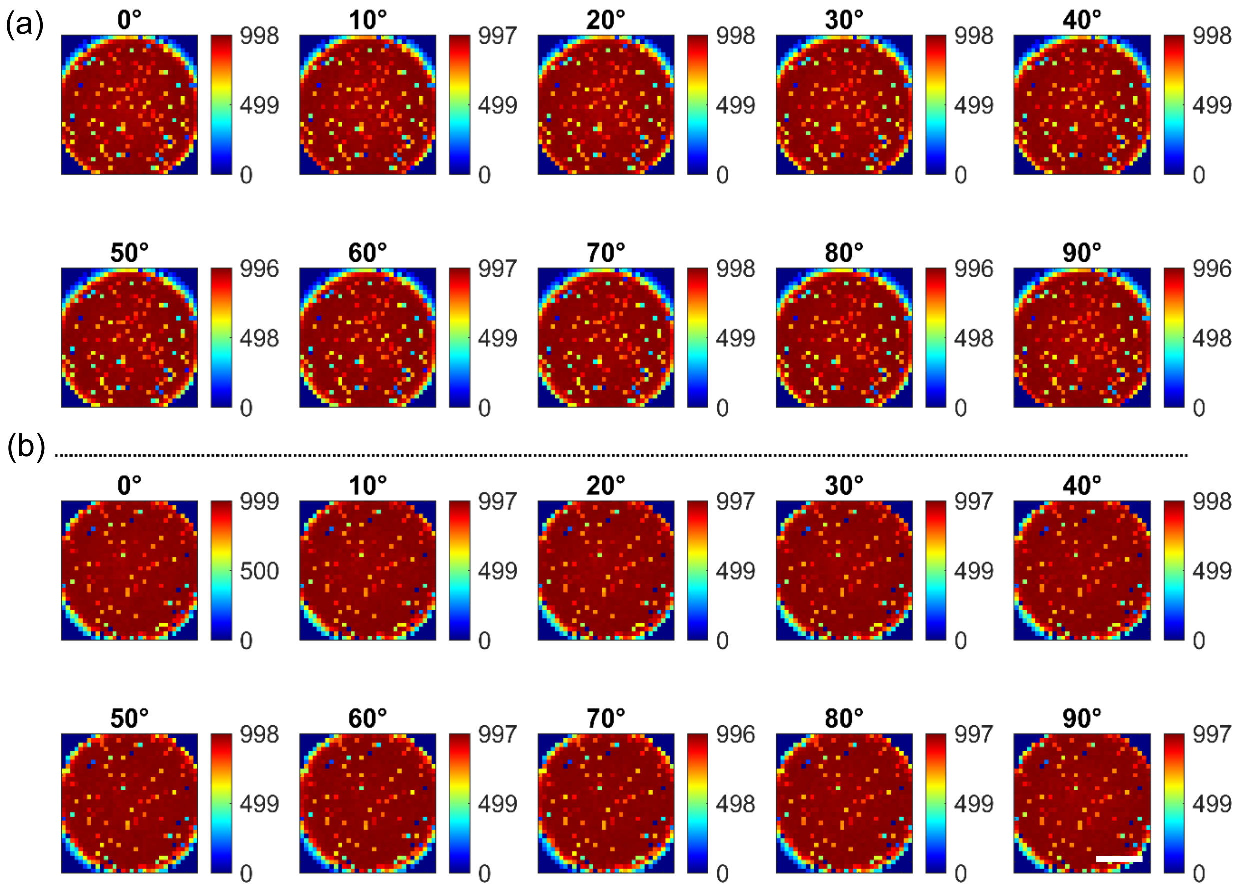


**Supplementary Figure S7: Topography of summed photon counts across QWP angles for multimode fiber output**. Topography of summed photon counts across 10 QWP angles for SPAD array 1 (a) and SPAD array 2 (b) for MMF. Scale bar, 0.5 mm.

In a graded-index MMF, each pixel records a different speckle grain—a coherent sum of many modes with slightly different delays and polarization states. Consequently, rotating the QWP does not produce a simple two-mode power exchange (as in the FMF case); instead, it subtly reweighs a high-dimensional modal mixture, yielding small, pixel-specific changes. In Supplementary Figure S8a, the individual plots are normalized per-angle, so global power variations are removed, and the traces mainly reflect redistribution of power across pixels and between the two analyzer channels; a shallow modulation is therefore expected. Supplementary Figure S8b (deviation from each pixel's mean) highlights these modest, angle-dependent changes as the familiar 'fanned-out' envelopes. This behaviour is typical of MMF speckle polarimetry and contrasts with the pronounced, quasi-sinusoidal modulation observed in few-mode fiber case.

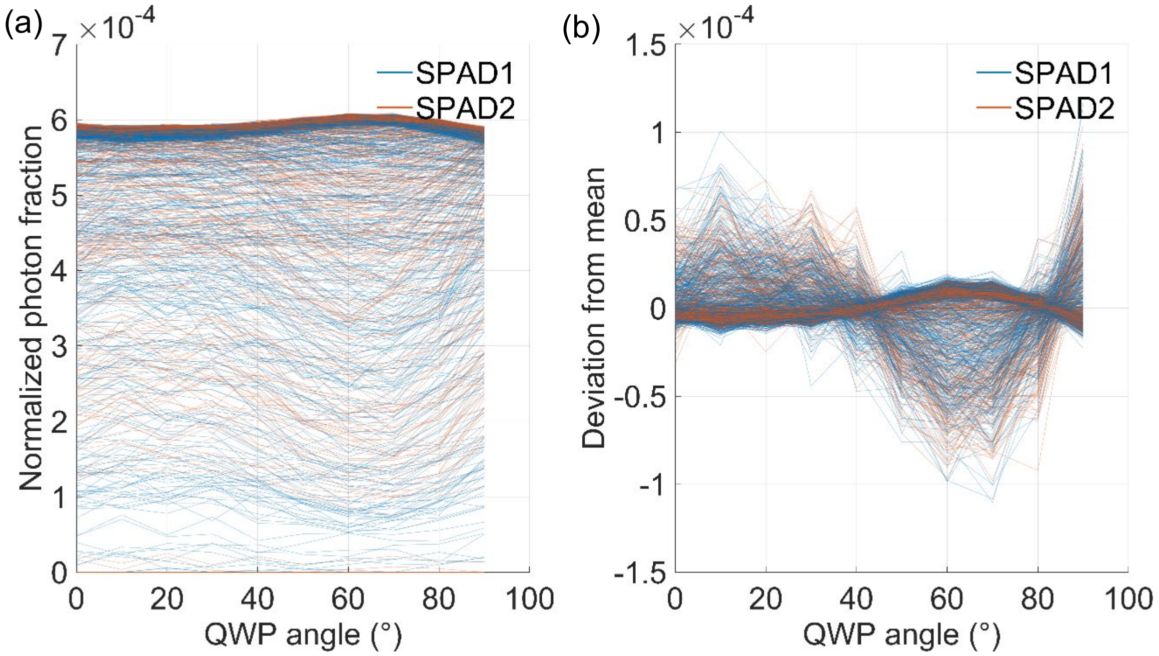


**Supplementary Figure S8: Polarization-dependent photon fractions across QWP angles for multimode fiber output.** (a) Normalized photon fractions recorded across 10 QWP angles for two orthogonal polarization channels (SPAD1 – V: blue, SPAD2 – H: red) for MMF. Each trace corresponds to one of the 932 SPAD pixels per array after the mask is applied. Panel (b) shows the deviation of each pixel's photon count from its mean across angles, revealing the polarization modulation behavior.

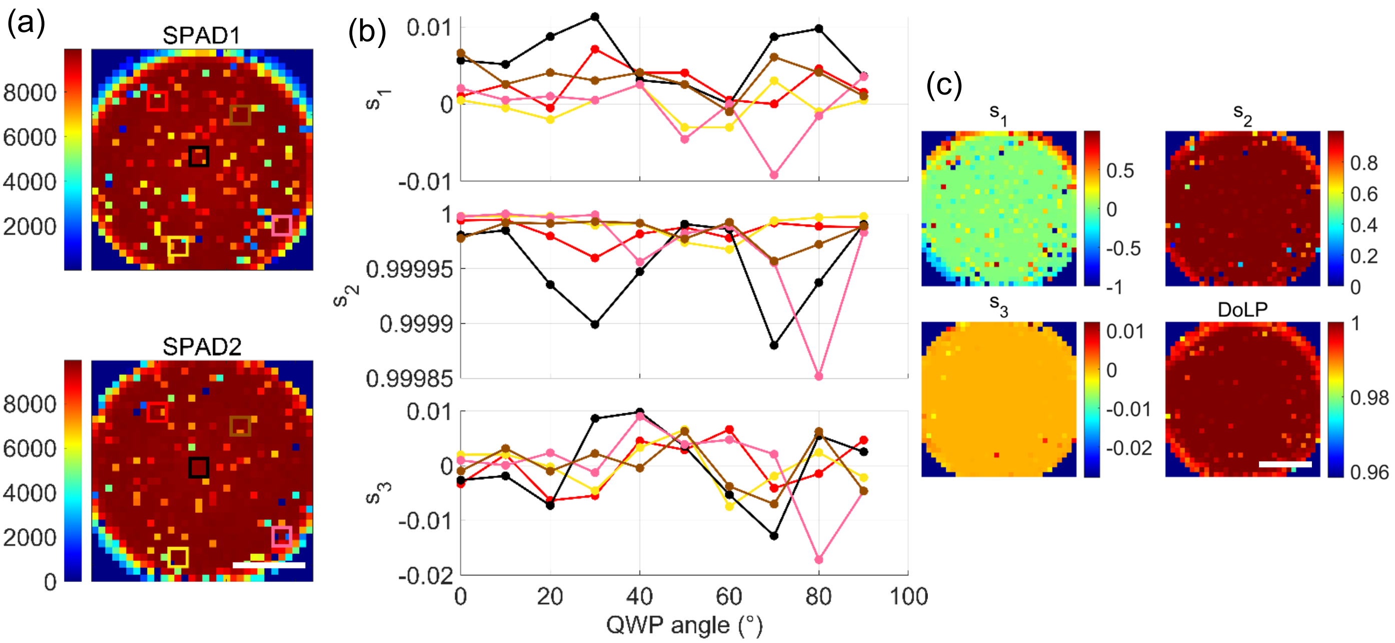


**Supplementary Figure S9: Stokes vector analysis for MMF.** (a) Topography of summed photon counts over 10 QWP angles across the SPAD arrays. (b) Stokes vectors for 5 representative pixels (colored boxes in a). (c) Mean $s_1$, $s_2$, $s_3$, and DoLP, showcasing the global Stokes obtained from average values over 10 QWP angles. Scale bars, 0.5 mm.

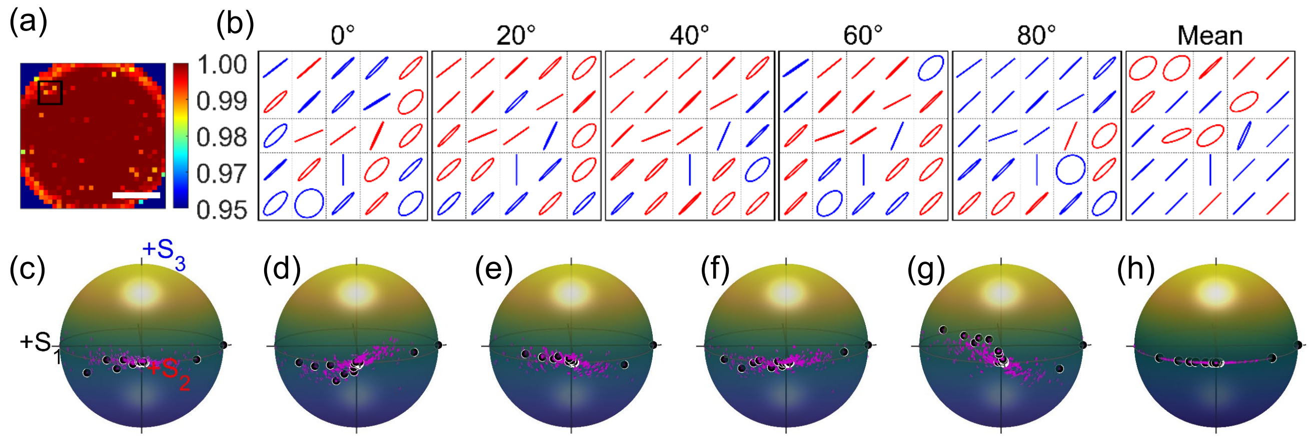


**Supplementary Figure S10: Polarization characterization and Poincaré representation in MMF.** (a) Mean degree of polarization across the 32×32 array; the black box indicates the selected 5×5 region for detailed analysis. Scalebar, 0.5 mm. (b) Polarization ellipses for each representative pixel in the selected region at QWP angles of 0°, 20°,40°,60°, 80°and the mean over all angles. Ellipse orientation and eccentricity encode the polarization azimuth and ellipticity, while color indicates handedness (blue = right-handed, red = left-handed). (c-g) Poincaré spheres showing the distribution of Stokes vectors across the full array for QWP angles 0°, 20°,40°,60°, and 80. (h) Mean Stokes vectors over all angles, summarizing the overall polarization state.

## Supplementary Note 4: Description of Supplementary Movie 3

**Animation summary**: The animation reveals spatio-temporal dynamics in a graded-index MMF. Each SPAD pixel records a single speckle grain—i.e., a coherent mix of many fiber modes—with slightly different delays and polarization states. As the 55-ps analysis bin sweeps along the TCSPC trace, the modal composition within the bin evolves: early-arriving modes drop out, later modes enter, and their relative phases drift. Consequently, each pixel's intensity shifts in time, and its Stokes vector rotates on the Poincaré sphere.

**Origin of the modest modulation**: In this MMF, the graded-index core reduces intermodal dispersion, so changes in modal delay are modest. Weak mode coupling and residual birefringence further modulate the output, producing slow rotations and occasional small steps in polarization. Launch conditions (input state of polarization and spot size) determine which modes are exciting, leading to pixel-dependent trajectories. Finite spectral bandwidth and the instrument response primarily smooth fine structure and can lower the apparent degree of polarization. Overall, the behaviour reflects genuine, time-resolved interference among mode groups in the fiber.

**Partial rotations**: The Stokes trajectories appear as partial arcs rather than full rotations across the 1-ns window. This is expected and can arise from several factors: in a graded-index MMF, the relative phase between dominant mode groups may not traverse a full $2\pi$ within the window; a steady bias in the coherent modal sum can anchor the trajectory; and the 55-ps gate, together with IRF smoothing, shortens the apparent path on the Poincaré sphere. The 0°–90° QWP scan sets the launch states and may further constrain the accessible rotation, though it is not the primary limiter for the time-sweep shown.

### Supplementary Note 5: Pixel-wise sensitivity and SNR calculations

We quantify sensitivity as minimum detectable modulation (MDM) from six repeated measurements under a fixed linear polarizer (both SPAD arrays illuminated by the same fiber output; laser-blocked and backgrounds subtracted). For each pixel in the 32×32 arrays, the background-corrected repeat standard deviation defines variability; MDM = 3σ / mean (fractional, 3σ). To avoid bias from a tiny hot-pixel tail, we apply a one-sided upper-tail mask (hot-pixel/outlier removal); no low-end clipping is used. Plots show topographic maps (left) and histograms (right); analysis 2-ms acquisition per measurement within an ROI.

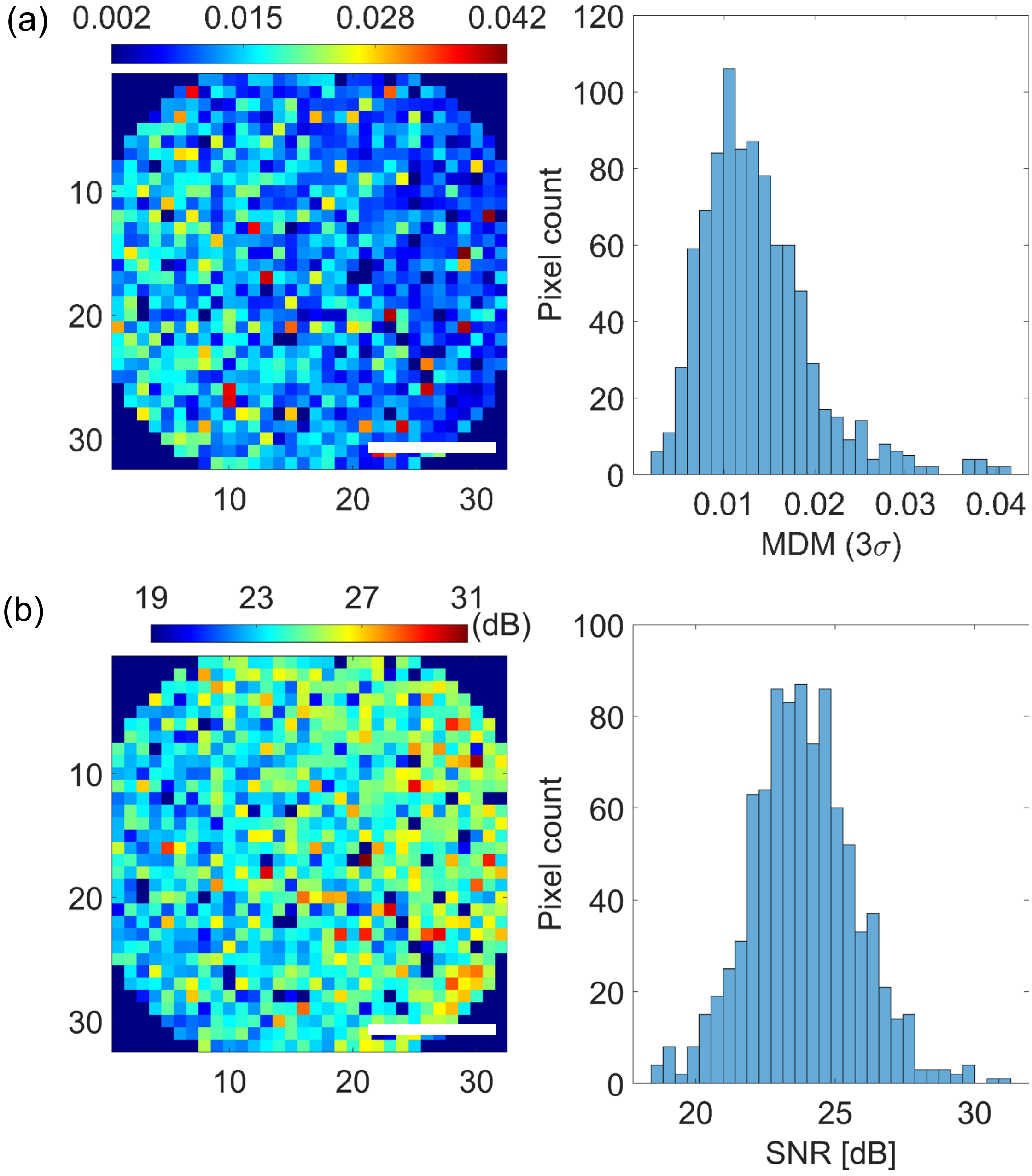


**Supplementary Figure S11: Sensitivity and signal-to-noise ratio for SPAD array 1.** (a) Per-pixel minimum detectable modulation (MDM, 3σ; unitless fraction). (b) Per-pixel SNR (dB). Left panels show topographic maps; right panels show the corresponding histograms. Scale bars, 0.5 mm.

After outlier removal (28 pixels for both arrays), SPAD1: MDM = 0.0023–0.030 (mean ≈ 0.013); SPAD2: 0.0016–0.023 (mean ≈ 0.0125), corresponding to per-pixel SNR ≈ 31–19 dB (SPAD1) and 33–21 dB (SPAD2). Thus, the system resolves ~0.2% modulation on single pixels while maintaining ~1.3% array-average sensitivity.

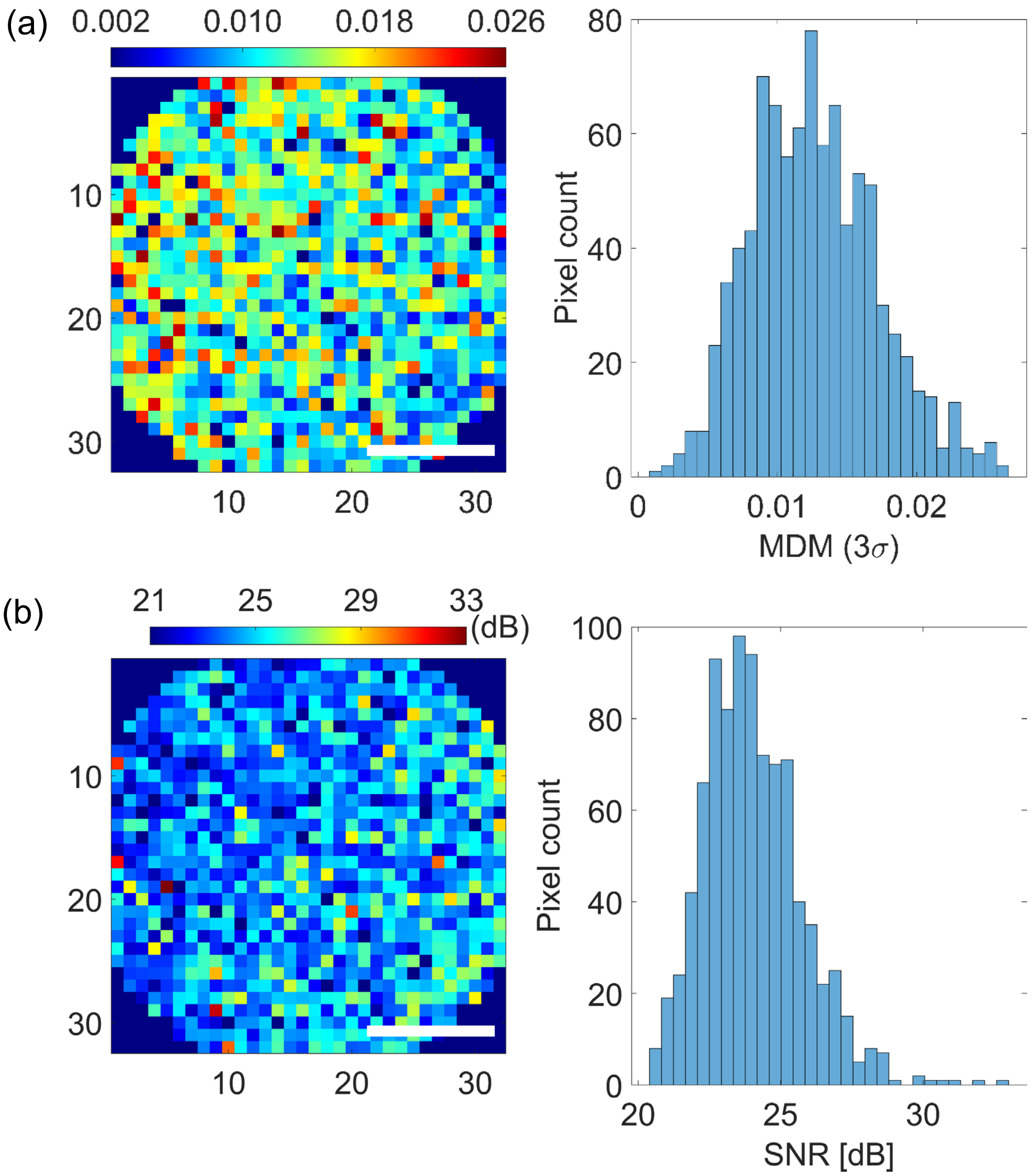


**Supplementary Figure S12: Sensitivity and signal-to-noise for SPAD array 2.** (a) Per-pixel minimum detectable modulation (MDM, 3σ; unitless fraction). (b) Per-pixel SNR (dB). Left panels show topographic maps; right panels show the corresponding histograms. Scale bars, 0.5 mm.

**Supplementary Note 6: Stability and drift characterization**

To quantify the stability of the dual-SPAD polarimetry system, we computed the coefficient of variation (CV) per pixel from the same repeated, background-subtracted measurements used for MDM. Assessing CV-based stability together with drift (percent change per repeat from a linear fit) is essential to show that the reported sensitivity (MDM) and SNR reflect true instrument performance rather than short-term noise or slow temporal wander. These checks confirm that pixel responses are repeatable and free of systematic trends over the acquisition, ensuring that the observed polarization-dependent modulations are real and reproducible.

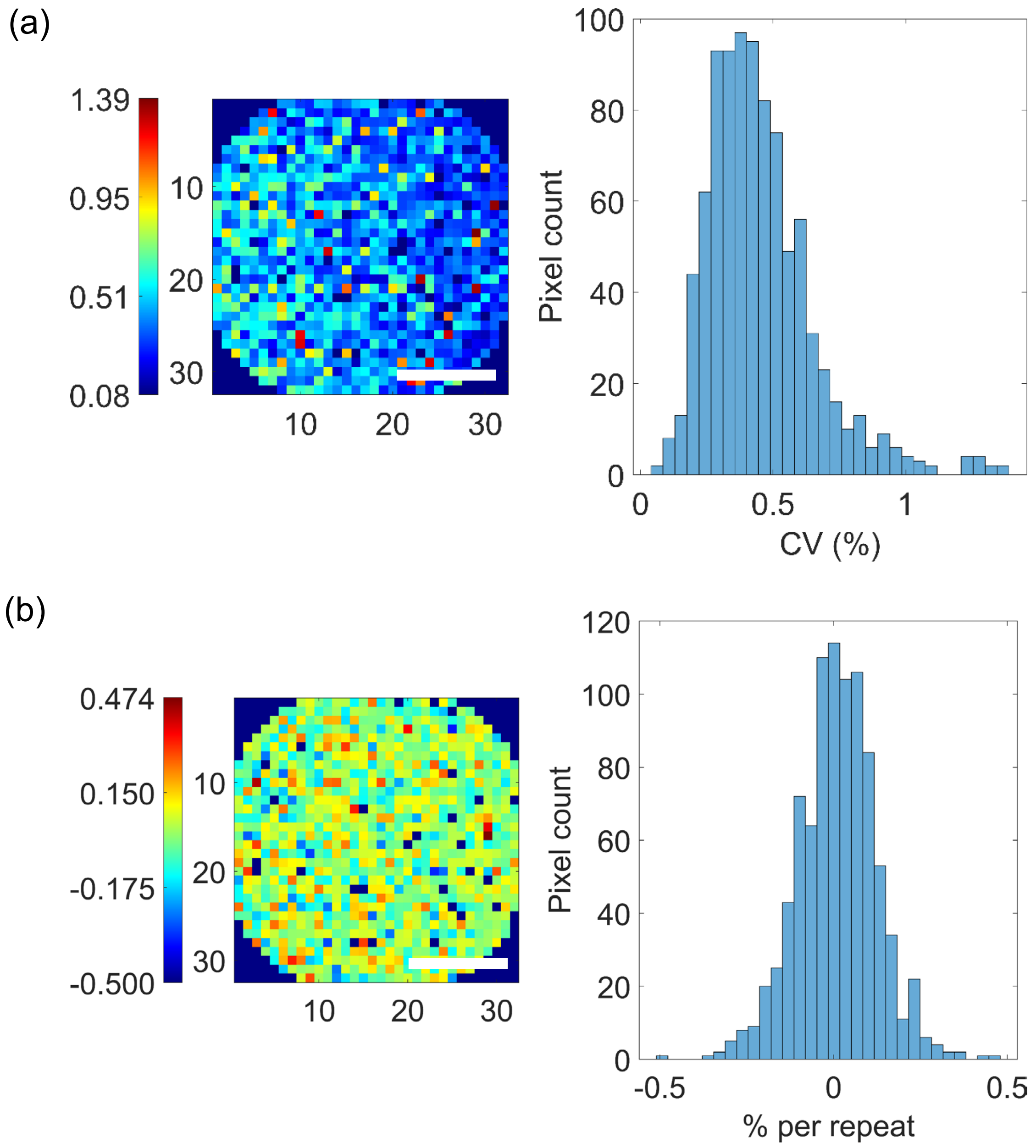


**Supplementary Figure S13: Stability and drift measurements for SPAD array 1.** (a) Per-pixel short-term stability, shown as CV (coefficient of variation) computed from the repeated measurements. (b) Drift map, reported as percent change per repeat from a linear fit to each pixel's repeat series. Left panels show spatial maps; right panels show the corresponding histograms. Scale bars, 0.5 mm.

Both SPAD arrays show low short-term variability: SPAD array 1, mean CV - 0.45% (interquartile range (IQR) 0.245%, 95th 0.841%) and SPAD array 2, mean CV - 0.42% (IQR 0.214%, 95th 0.699%). Interpreting CV in the same framework as our sensitivity, MDM is about three times CV, giving ~1.27% for SPAD array 1 and ~1.25% for SPAD array 2; using SNR as 1/CV gives ~23.7–23.8 dB. Mean drift is +0.01% per repeat for SPAD array 1 and −0.007% per repeat for SPAD array 2; over six repeats (about five intervals)—an order of magnitude smaller than the CV. In practical terms, the system is stable, repeatable, and not drift-limited over the measurement window, which strengthens the credibility of the ultrafast polarimetry results.

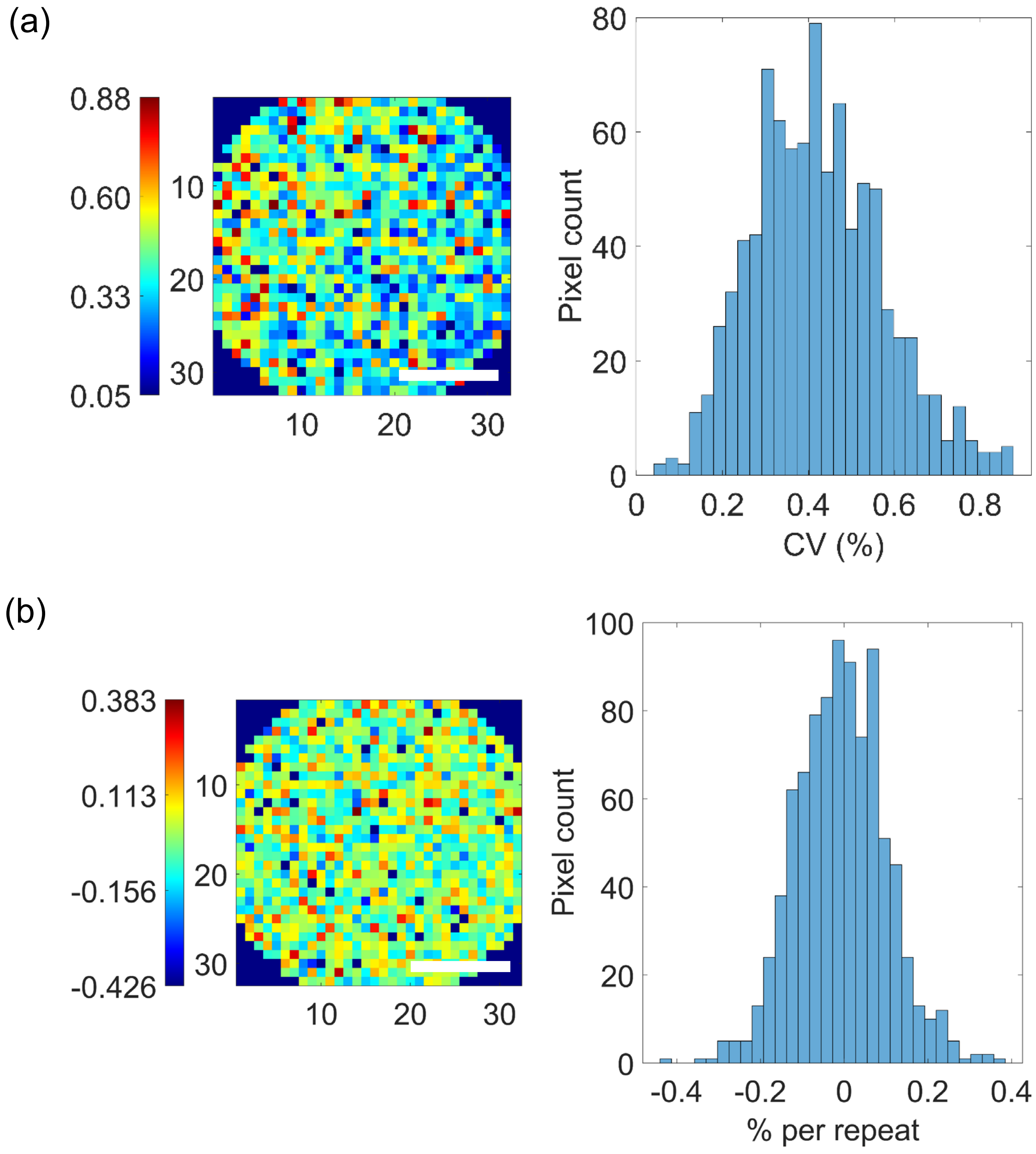


**Supplementary Figure S14: Stability and drift measurements for SPAD array 2.** (a) Per-pixel short-term stability, shown as CV computed from the repeated measurements. (b) Drift map, reported as percent change per repeat from a linear fit to each pixel's repeat series. Left panels show spatial maps; right panels show the corresponding histograms. Scale bars, 0.5 mm.

**Supplementary Note 7: Instrument response function characterization of the SPAD arrays**

To assess the temporal performance of the SPAD detectors, the instrument response function (IRF) of every pixel in both arrays was measured under uniform illumination from a narrow-band pulsed laser at 852 nm delivered through a PMF. The IRF reflects the combined timing broadening resulting from the excitation pulse duration, detector electronic jitter, and additional jitter introduced by RF cables and associated electronics. For each pixel, the corresponding TCSPC histogram was recorded and its full width at half maximum (FWHM) extracted to quantify the IRF.

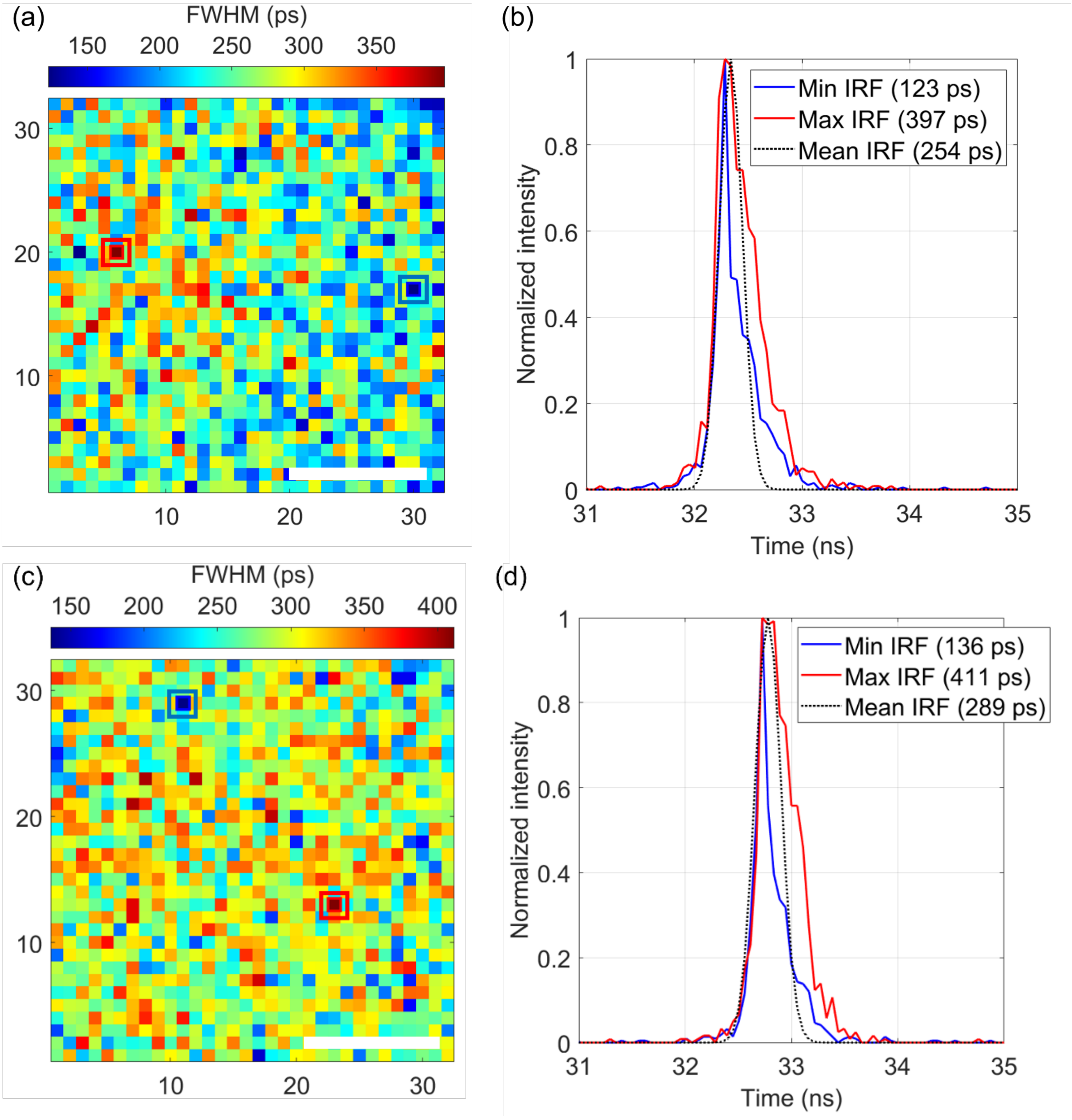


**Supplementary Figure S15: Instrument response function characterization of the SPAD arrays.** (a) Topography of the IRFs for SPAD array 1, shown as the FWHM of the measured TCSPC histograms. Pixels exhibiting the minimum and maximum IRF values are highlighted with blue and red rectangular markers, respectively. (b) Corresponding temporal traces for these pixels, together with a simulated Gaussian IRF (black) constructed using the mean FWHM across the array. (c–d) Same measurements for SPAD array 2. Scale bars, 0.5 mm.

Supplementary Figure S15 summarizes the results. Supplementary Figures S15a and S15c show the IRF topography maps for SPAD arrays 1 and 2, respectively, where each pixel is represented by the FWHM of its measured temporal response. Across the array, the IRF FWHMs ranged from 123 to 398 ps for SPAD array 1 (253 ± 48 ps) and 136–411 ps for SPAD array 2 (289 ± 42 ps). Supplementary Figures S15b and S15d present representative IRF traces from the pixels exhibiting the minimum and maximum measured FWHM, together with a simulated Gaussian IRF corresponding to the mean FWHM across each array. These measurements provide a direct visualization of the spatial variation in timing performance across the arrays and establish the baseline temporal characteristics used throughout the main experiments.
