# Supplementary material for: Mode-resolved picosecond single-photon polarimetry maps modal dynamics in multimode fibers
Source: Nat Commun. 2026 May 7;17:6174. doi: 10.1038/s41467-026-72129-w (PMC13365444; doi:10.1038/s41467-026-72129-w)
Supplement: Supplementary file 2 — Description of Additional Supplementary Files [file 41467_2026_72129_MOESM2_ESM.pdf]

Description of Additional Supplementary Files

Supplementary Movie 1: Real-time intensity evolution of LP_{01} (top) and LP_{11} (bottom) spatial modes recorded simultaneously on two SPAD arrays during controlled cantilever-induced strain. The left panels correspond to SPAD1 (vertical polarization channel) and the right panels to SPAD2 (horizontal polarization channel). Each frame represents one of 16 sequential strain points, illustrating polarization-resolved energy flow and mode-profile evolution across the two orthogonal polarization states.

Supplementary Movie 2: Spatial maps of the Stokes parameters s_1, s_2, and s_3 reconstructed across the SPAD arrays. The left panels show LP_{01} and the right panels LP_{11}. Each frame corresponds to a single QWP angle (0°–90°), illustrating the per-pixel evolution of the Stokes parameters with specific waveplate rotation.

Supplementary Movie 3: Time-resolved Stokes-vector evolution in a graded-index multimode fibre. The top panels show representative TCSPC histograms from the same spatial pixel recorded on SPAD1 and SPAD2, corresponding to the vertical and horizontal polarization channels. Bottom panels display the full scan across the TCSPC trace (61 temporal bins), illustrating the evolution of reconstructed Stokes vectors for QWP angles 0°, 40°, and 90°. Black markers represent Stokes vectors obtained from the selected 5 × 5 spatial region, while pink points correspond to Stokes vectors from the remaining spatial locations across the array.
