# Supplementary material for: Mode-resolved picosecond single-photon polarimetry maps modal dynamics in multimode fibers
Source: Nat Commun. 2026 May 7;17:6174. doi: 10.1038/s41467-026-72129-w (PMC13365444; doi:10.1038/s41467-026-72129-w)
Supplement: Supplementary file 6 — Transparent Peer Review file [file 41467_2026_72129_MOESM6_ESM.pdf]

nature portfolio

Peer Review File

Mode-resolved picosecond single-photon polarimetry maps modal dynamics in multimode fibers

Corresponding Author: Dr Harikumar K Chandrasekharan

This file contains all reviewer reports in order by version, followed by all author rebuttals in order by version.

Version 0:

Reviewer comments:

Reviewer #1

(Remarks to the Author)
The paper by Chandrasekharan and Donaldson reports on the measurements of polarimetric modal dynamics in multimode optical fibers using time-resolved SPAD array. Although the technology is very useful and the paper is well written, the overall evaluation of the work is incremental, compared with previously existing work in the field, as summarized from Line 69-Line 78. The success of the experimental implementation mostly builds upon the use of the silicon CMOS SPAD array.

A few specific, technological questions:

1) How did the authors measure the Stokes vectors, as presented in Fig. 5? Were they measured by the experimental setup presented in Fig. 1 (a)? It is unclear to me.

2) As many time-resolved measurements are relevant to the IRF of the photon counting system, including the SPAD and the time tagger, I suggest measure and present the IRF in the supplementary information with one of the SPADs in the array.

(Remarks on code availability)

Reviewer #2

(Remarks to the Author)
The authors present a polarisation resolved method for characterising the spatial mode dynamics in a few mode fibre with 55ns temporal resolution using a tandem of SPAD arrays, with 1,000 pixels resolution each, and showcase the system under various perturbations of the fibre. They also characterise the polarisation dynamics in a highly multimode fibre. Although the full spatial characterisation is not possible in the current system for that case, which I understand, their data reveals how different modal composition in a given time arrival interval affect the polarisation state, which I find very interesting on its own. The paper is well structured and written and I was impressed with how carefully the experiments very designed and performed and how well the data was analysed. It is rare to see high-quality science like this. If the authors can correct and comment on the following points below, I would be happy to recommend this paper to be accepted.

- is there some way to resolve the temporal dynamics of the near-degenerate modes using some form of spatial filter or correlation filter, such as using methods in the following papers (Opt. Lett. 37, 2478 (2012); Opt. Express 17, 9347 (2009), Nat Comms 13, 4294 (2022)?
- I noticed that each SPAD chip has a few dead pixels. Can you please comment how this affects the measurement, especially the local polarisation measurement? Is the polarisation state discarded for dead pixels or some interpolation applied?
- in Fig 2a (middle) I was surprised to see the fundamental mode so squeezed (much smaller than the waist of the fundamental mode) and I am curious what mechanism cause such behaviour? Can you comment on this please if possible?
- Figure 5 (e-h) fix "analyses" to "analysis"?
- how sensitive is the setup to relative offset of the two SPAD chips? You mention array partitioning helps with their overlap but I am not sure how it works. Can you explain it a bit more in the paper? Also, what happens if one of the SPAD chips is

tilted?
- you mention that 41 spatial modes can be resolved in a 3.2km fibre but obviously if I make the fibre longer, the modes will get more separated with respect to each other in temporal domain due to different propagation constants, and I will be able to resolve more of them. What is the reason to choose 3.2 km for length? Any practical reason? It feels random while reading the paper so consider providing some more nuanced arguments.
- Line 440. The sentence "In contrast, With"-> fix to lower case "with".

(Remarks on code availability)

Version 1:

Reviewer comments:

Reviewer #2

(Remarks to the Author)
I thank the authors for addressing all my questions and concerns and for their detailed replies that made the concepts presented in the paper clearer to me. I recommend the manuscript to be published as is.

(Remarks on code availability)

Open Access This Peer Review File is licensed under a Creative Commons Attribution 4.0 International License, which permits use, sharing, adaptation, distribution and reproduction in any medium or format, as long as you give appropriate credit to the original author(s) and the source, provide a link to the Creative Commons license, and indicate if changes were made.
In cases where reviewers are anonymous, credit should be given to 'Anonymous Referee' and the source.
The images or other third party material in this Peer Review File are included in the article’s Creative Commons license, unless indicated otherwise in a credit line to the material. If material is not included in the article’s Creative Commons license and your intended use is not permitted by statutory regulation or exceeds the permitted use, you will need to obtain permission directly from the copyright holder.
To view a copy of this license, visit https://creativecommons.org/licenses/by/4.0/

Point-by-Point Response to Reviewers:

Mode-resolved picosecond single-photon polarimetry maps modal dynamics in multimode fibers

We thank the reviewers for their time and effort in evaluating our manuscript. Below, we provide point-by-point responses to each comment, with the reviewers' remarks shown in **bold** and our replies in regular text. All corresponding revisions have been incorporated into the updated manuscript.

Before addressing the specific comments, we also highlight several corrections and improvements made independently of the reviewers' feedback:

1. Duplicate citations [11] and [21] have been corrected.
2. The spectral range of the SPAD arrays—previously omitted—has now been added (page 4, line 131).
3. Supplementary Movie 2 has been updated: the previous version included only the angle-resolved Stokes animation for the LP_{01} mode. The revised movie now also includes the LP_{11} mode, providing a more complete mode-resolved Stokes representation.

Reviewer #1

The paper by Chandrasekharan and Donaldson reports on the measurements of polarimetric modal dynamics in multimode optical fibers using time-resolved SPAD array. Although the technology is very useful and the paper is well written, the overall evaluation of the work is incremental, compared with previously existing work in the field, as summarized from Line 69-Line 78. The success of the experimental implementation mostly builds upon the use of the silicon CMOS SPAD array.

Response:

We thank the reviewer for the positive assessment of our work and for the opportunity to clarify the novelty and contributions of this study.

The contribution of this work does not lie in the use of CMOS SPAD arrays alone, but in the new measurement architecture, reconstruction methodology, and polarimetric capabilities enabled by the overall system.

First, the dual-SPAD configuration provides simultaneous, pixel-wise detection of the two orthogonal polarization components across ~1000 spatial channels, allowing direct observation of picosecond-scale polarization-resolved mode dynamics. These

capabilities are not accessible with balanced detection, sequential scanning, or camera-based approaches, all of which lack true simultaneity, picosecond temporal sampling, or single-photon sensitivity.

Second, by inserting a single QWP before PBS, the same setup performs full Stokes reconstruction with 55-ps temporal sampling across all pixels without mechanical scanning or multiple analyzer states. This produces ultrafast, spatially continuous, and photon-efficient full-field polarimetry.

Third, in contrast with prior methods such as DoFP/DoAP polarimetry, speckle-based Stokes imaging, interferometric MMF characterization, and SNSPD-based Stokes systems, our approach uniquely combines simultaneous spatial, temporal, modal, and polarization resolution at the single-photon level. To our knowledge, no prior method provides picosecond-resolved, mode-specific Stokes vectors or scan-free dual-polarization detection in MMFs or FMFs.

Fourth, our Hilbert-transform Stokes estimator constitutes a methodological advance beyond conventional fitting-based polarimetry. Traditional systems recover Stokes parameters by fitting intensity versus analyzer angle to sinusoidal or Fourier models, yielding only *global* polarization estimates and masking spatially localized behavior—particularly in multimode fields with anti-symmetric structure. In contrast, the Hilbert approach extracts the instantaneous *amplitude and phase of the H and V components at each pixel and each QWP angle without global curve fitting.* This enables per-pixel, per-angle Stokes retrieval under photon-limited conditions and reveals features that are otherwise averaged out. This analytic-signal method is essential for capturing the spatially structured, ultrafast polarization dynamics intrinsic to few-mode and multimode propagation, and, to our knowledge, has not been demonstrated previously for MMF polarimetry.

These combined advances—simultaneous dual-polarization single-photon detection, picosecond sampling, full-field scan-free Stokes retrieval, and Hilbert-based per-pixel Stokes estimation—define a new measurement regime, rather than an incremental improvement over existing systems.

As the reviewer notes, the resulting platform is broadly useful and enables applications we discuss in the manuscript, including ultrafast MMF characterization, high-dimensional quantum photonics, and translational clinical tools. In particular, the same architecture naturally supports single-ended operation for imaging-guided interventions: any deformation of a surgical fiber probe would manifest directly as localized changes in the measured Stokes signatures.

To better communicate the novelty of the developed system, we have refined the introductory paragraph, fully complying with the permitted word limit (page 3, lines 79-95),

“In this paper, we present............ low-light MMF polarimetric imaging.”

We hope this clarification addresses the reviewer’s concerns regarding novelty and the distinct contributions of our approach.

Comment 1:
“How did the authors measure the Stokes vectors? Were they measured by the experimental setup in Fig. 1(a)?”

Response:
The Stokes vector measurements in this work were obtained using the same experimental setup shown in Fig. 1(a), with only a single modification: a quarter-wave plate was inserted before the PBS, and phase modulation was achieved by rotating the PBS (pages 9–10). This minimal addition was sufficient to demonstrate the feasibility of high-level, mode-resolved polarimetric characterization. We had this information in the draft (page 8, lines 246,247)

“……. enabled by the addition of a single optical component. Specifically, QWP was inserted before the PBS……”

To make this very clear for the reader, we have now added this information and modified the text as follows (page 8-9, lines 245,248),

“To demonstrate this capability, we made no changes to the existing setup in Figure 1a other than inserting a single additional optical element - a quarter waveplate (QWP)- before the PBS. This simple addition enables full reconstruction of the Stokes vector at each pixel in both polarization-resolved detection arms.”

Comment 2:
“….. I suggest measure and present the IRF in the supplementary information with one of the SPADs in the array..”

Response:
We thank the reviewer for this valuable suggestion. We have included the topography of the measured IRFs of both SPAD array pixels used in the work, as well as representative pixels with the smallest and largest IRFs, in the Supplementary Material (Note 7). These are referenced in the Methods section (highlighted in the revised draft, lines 498-511, pages 16-17).

Reviewer #2

The authors present a polarisation resolved method for characterising the spatial mode dynamics in a few mode fibre with 55ns temporal resolution using a tandem of SPAD arrays, with 1,000 pixels resolution each, and showcase the system under various perturbations of the fibre. They also characterise the polarisation

dynamics in a highly multimode fibre. Although the full spatial characterisation is not possible in the current system for that case, which I understand, their data reveals how different modal composition in a given time arrival interval affect the polarisation state, which I find very interesting on its own. The paper is well structured and written and I was impressed with how carefully the experiments very designed and performed and how well the data was analysed. It is rare to see high-quality science like this. If the authors can correct and comment on the following points below, I would be happy to recommend this paper to be accepted.

Response:

We thank the reviewer for the positive and encouraging evaluation of our work. We appreciate the recognition of our experimental design and analysis, and we have addressed all points raised with the appropriate clarifications and revisions.

Comment 1:
“is there some way to resolve the temporal dynamics of the near-degenerate modes using some form of spatial filter or correlation filter……?”

Response:
While our present system reports the combined behavior of the near-degenerate $LP_{11a/b}$ modes, we note that their temporal dynamics can indeed be resolved with minimal modification to the setup in Fig. 1. Specifically, inserting a free-space *correlation filter*—implemented as a static diffractive optical element (DOE) or phase plate matched to the LP_{11} basis functions—would spatially separate the two orientations before detection, enabling degeneracy-resolved picosecond tracking. Although the cited works (*Opt. Lett. 37, 2478 (2012); Opt. Express 17, 9347 (2009), Nat Comms 13, 4294 (2022)* often use adaptive optics such as SLMs, the same mode-selective projections can be implemented far more compactly and robustly using fixed free-space diffractive optical elements (DOEs). These DOEs integrate directly into our existing imaging path without requiring any changes to the SPAD architecture, enabling mode-selective and degeneracy-resolved measurements in MMFs and providing a clear route to further enhancing the novelty and impact of the system.

To inform this, we added the following in the draft (lines 160-161, page 4),

Future implementations could resolve the LP_{11}a/b orientations by inserting a simple correlation filter or phase mask.

Comment 2:
“- I noticed that each SPAD chip has a few dead pixels. Can you please comment how this affects the measurement, especially the local polarisation measurement?”

Response:
As part of our detector characterization, we examined all pixels under uniform 852-nm

pulsed illumination. Every pixel exhibited a measurable photon-response peak in its TCSPC trace, and no true dead pixels were identified. We did observe ~6% high-DCR pixels, which remain functional but exhibit elevated noise floors. Because these pixels still detect photons and because their number is small relative to the full array, the polarization-resolved measurements presented in the manuscript are unaffected. For completeness, we verified that excluding these pixels in post-processing does not alter the reported results. If needed, such pixels can be masked, interpolated, or excluded in post-processing, but no exclusion was required for the present work, as > 90% of the pixels were fully functional.

Comment 3:
"- in Fig 2a (middle) I was surprised to see the fundamental mode so squeezed …… Can you comment on this please if possible?"

Response:
We thank the reviewer for highlighting this point. The "squeezed'' appearance of the LP_{01} mode in the two-turn case of Fig. 2(a) arises from the strong, localized bend applied to the fiber. Such curvature induces asymmetric refractive-index perturbations that shift the field toward the inner bend and compress the mode-field diameter—a well-established behaviour in bent fibers (Marcuse, JOSA 1976; Snyder & Love, *Optical Waveguide Theory*, 1983). In our few-mode fiber, the effect is further enhanced by stress-induced coupling between LP_{01} and LP_{11} at the perturbation point ~2 m from the launch, where the modal fields are fully overlapped. This combined influence of bend-induced index gradients and stress-driven mode mixing leads to the reduced waist observed in Fig. 2(a).

Comment 4:
"Fix 'analyses' to 'analysis'."

Response:
Corrected.

Comment 5:
"- how sensitive is the setup to relative offset of the two SPAD chips? You mention array partitioning helps with their overlap but I am not sure how it works. Can you explain it a bit more in the paper?

Response:
We thank the reviewer for highlighting this important point. As noted in the manuscript (lines 137–143), the PF32 sensor regions are mounted using CS-thread adapters that allow the lens tubes to be rigidly secured directly to the PBS. Once assembled, the optical paths and sensor positions are fixed, and the system behaves as a mechanically unified module. In addition, irises placed at the PBS outputs (immediately before the sensors) ensure that any lateral or rotational misalignment would be immediately visible in real-time mode images during acquisition. In our experiments, no such relative offset was observed. We have not performed a dedicated sensitivity analysis to quantify the

effect of intentional offsets, but the system's mechanical stability and visual alignment checks ensured consistent overlap between the two imaging paths.

…."You mention array partitioning helps with their overlap but I am not sure how it works".

We have clarified in the revised manuscript (page 11, lines 335-343) how array partitioning mitigates the effect of any potential lateral or rotational offset between the two SPAD arrays. Specifically, the partitioning operates on regional averages rather than strict pixel-to-pixel correspondence, ensuring that the retrieved Stokes parameters remain robust even if the arrays probe slightly different parts of the modal field. Because the modal patterns vary smoothly, these regional averages are largely insensitive to subpixel misalignments, effectively filtering out any residual chip displacement. This explanation has now been incorporated into the main text to improve clarity.

….."Also, what happens if one of the SPAD chips is tilted?"

Concerning the possibility of one SPAD chip being tilted, such an effect can be calibrated and corrected. A standard approach is to project a calibrated test pattern (e.g., a USAF target) onto both arrays and recover the relative transformation using overlap-integral or edge-detection methods. The resulting transformation matrix (translation, rotation, or mild perspective correction) can then be applied during signal processing to register the two images before polarization reconstruction. Moreover, because the analysis can operate on regional averages rather than strict pixelwise mapping, small residual tilts or misregistration do not compromise measurement integrity.

Comment 6:
"**you mention that 41 spatial modes can be resolved in a 3.2km fibre but obviously if I make the fibre longer, the modes will get more separated with respect to each other in temporal domain due to different propagation constants, and I will be able to resolve more of them. What is the reason to choose 3.2 km for length? Any practical reason? It feels random while reading the paper so consider providing some more nuanced arguments**."

Response:
We thank the reviewer for raising this important point. The choice of a 3.2-km fiber length was not arbitrary. As clarified in the revised manuscript, the number of temporally resolvable modes is jointly limited by (i) the differential delay required to separate adjacent modes and (ii) the 56-ns dynamic range of the SPAD–TCSPC system. For the fiber parameters used here, 3.2 km provides a delay spread that allows up to 41 modes to be cleanly resolved while keeping the slowest mode within the 56-ns acquisition window. Extending the fiber length would push the latest-arriving modes beyond this window, causing them to wrap into earlier time bins—an inherent behavior of TCSPC systems—which would scramble and corrupt the TCSPC trace. We have now added the

following explanation to the manuscript (page 12, lines 352-357) to clarify why 3.2 km represents a practical limit for robust mode resolution with the present hardware.

This fiber length was selected to maximize the usable modal delay spread while ensuring that the slowest mode remains within the 56-ns acquisition window; beyond this range, later-arriving modes would fall outside the TCSPC window and wrap into the earliest bins, reappearing as full aliased peaks that overlap with the first-arriving modes and effectively merge those bins, scrambling the temporal-domain modal identification.

Comment 7:
“Fix capitalisation of ‘With’.”

Response:
Corrected.
